# Incorporation of apolipoprotein E into HBV–HCV subviral envelope particles to improve the hepatitis vaccine strategy

**DOI:** 10.1038/s41598-021-01428-7

**Published:** 2021-11-08

**Authors:** Elsa Gomez-Escobar, Julien Burlaud-Gaillard, Clara Visdeloup, Adeline Ribeiro E Silva, Pauline Coutant, Philippe Roingeard, Elodie Beaumont

**Affiliations:** 1 INSERM Unit 1259 MAVIVH, Université de Tours and CHRU de Tours, 10 boulevard Tonnellé Tours, Tours, France; 2 Plate-Forme IBiSA des Microscopies, PPF ASB, Université de Tours and CHRU de Tours, Tours, France

**Keywords:** Hepatitis C, Protein vaccines, Hepatitis C virus

## Abstract

Hepatitis C is a major threat to public health for which an effective treatment is available, but a prophylactic vaccine is still needed to control this disease. We designed a vaccine based on chimeric HBV–HCV envelope proteins forming subviral particles (SVPs) that induce neutralizing antibodies against HCV in vitro. Here, we aimed to increase the neutralizing potential of those antibodies, by using HBV–HCV SVPs bearing apolipoprotein E (apoE). These particles were produced by cultured stable mammalian cell clones, purified and characterized. We found that apoE was able to interact with both chimeric HBV–HCV (E1-S and E2-S) proteins, and with the wild-type HBV S protein. ApoE was also detected on the surface of purified SVPs and improved the folding of HCV envelope proteins, but its presence lowered the incorporation of E2-S protein. Immunization of New Zealand rabbits resulted in similar anti-S responses for all rabbits, whereas anti-E1/-E2 antibody titers varied according to the presence or absence of apoE. Regarding the neutralizing potential of these anti-E1/-E2 antibodies, it was higher in rabbits immunized with apoE-bearing particles. In conclusion, the association of apoE with HCV envelope proteins may be a good strategy for improving HCV vaccines based on viral envelope proteins.

## Introduction

About 71 million people worldwide are chronically infected with hepatitis C virus (HCV), a major cause of severe chronic liver disease that may progress to hepatocellular carcinoma (HCC)^1,2^. Therapies based on the use of direct-acting antivirals (DAAs) are available and highly effective (with cure rates above 95%), but with some limitations. Most infected individuals are asymptomatic, so only a small percentage are diagnosed and, therefore, treated. The cost of DAAs, which has decreased in recent years, is less of a problem than the cost of the screening logistics required to identify chronic carriers of HCV worldwide that have yet to be treated^3^. Moreover, reinfection can occur, because these drugs have no protective effect, and they do not fully prevent the development of severe liver diseases, such as HCC, either^4–6^. DAAs can also fail to cure infections involving several distantly related genotypes of HCV^7–9^. The World Health Organization (WHO) aims to eliminate viral hepatitis as a public health threat by 2030. However, it was estimated in 2015 that two million new HCV infections occur annually, which is much greater than the number of people cured (843,000 individuals)^1^. Recent studies have suggested that the WHO’s goals are likely to be reached only locally in a few countries, not globally^10,11^. In light of this evidence, it will be difficult to control the spread of HCV without a prophylactic vaccine.

The spontaneous clearance of HCV has been observed in 15–25% of patients and is related to early cellular and humoral responses^12–14^. This early induction of neutralizing antibodies (Nabs), together with a T-cell response, is the key to containing the infection and provides important leads for the development of a prophylactic vaccine. Nevertheless, a randomized clinical trial phase 1/2 (NCT01436357) resulted in the induction of T cell responses but did not prevent the development of chronic infection in comparison to the placebo group, which may suggest that B cell responses may be essential in the prevention of the disease progression^15^. In fact, most of the preventive vaccines against various pathogens currently available are based on the development of Nabs^16^. In the case of HCV, the envelope glycoproteins (E1 and E2) are the natural targets of these antibodies and have been widely used in various prototype vaccines^17–21^. One of these candidate vaccines, based on recombinant HCV E1 and E2 proteins, was shown to elicit high titers of anti-HCV envelope antibodies with cross-neutralizing properties, together with a T-cell response, in a human phase I clinical trial (NCT00500747)^17,22^. However, the E1 and E2 glycoproteins are transmembrane proteins and are thus difficult to extract and purify in large-scale manufacturing processes. We have tried to overcome this problem by developing a prototype HCV vaccine consisting of full-length HCV E1 or E2 glycoproteins (from genotype 1a) fused to the heterologous hepatitis B virus (HBV) small envelope protein (S), also known as small hepatitis B surface antigen (SHBsAg), that self-assemble into highly immunogenic, noninfectious and secreted subviral particles (SVPs) resembling the HBV vaccine^23^. We have shown that these particles can induce strong specific antibody responses directed against the HCV and HBV envelope proteins in immunized rabbits, together with Nabs capable of neutralizing HCV infection in vitro^24,25^. Moreover, we have shown that the E1 and E2 proteins are more immunogenic when presented separately in the SVPs^26^. Finally, we recently reported that immunization with mixtures of SVPs presenting HCV envelopes of different genotypes improves the induction of cross-neutralizing antibodies against various genotypes of HCV^27^.

However, it is important to take the close interactions of HCV with host lipid metabolism into account when developing a vaccine against this virus. HCV associates with lipid components and circulates in the bloodstream of infected patients as lipoviral particles (LVPs), which help the virus to escape from Nabs^28^. Apolipoprotein E (apoE) is a component of HCV LVPs that has been shown to play a key role in the modulation of Nab sensitivity^29^. ApoE is a polymorphic lipoprotein that not only interacts with HCV envelope glycoproteins, but also participates in various stages of the viral cycle: entry, assembly and the maturation of infectious virions^30–33^. Consequently, apoE seems to be an important element for ensuring the correct folding and presentation of HCV envelope proteins as on the viral surface. In this study, we aimed to improve our vaccine model by reproducing the interactions between HCV envelope glycoproteins and apoE at the surface of HBV–HCV SVPs, with the ultimate objective of increasing the neutralizing properties of the antibodies induced by immunization.

## Results

### Effective interaction between apoE and chimeric HBV–HCV envelope proteins

**Figure 1:**
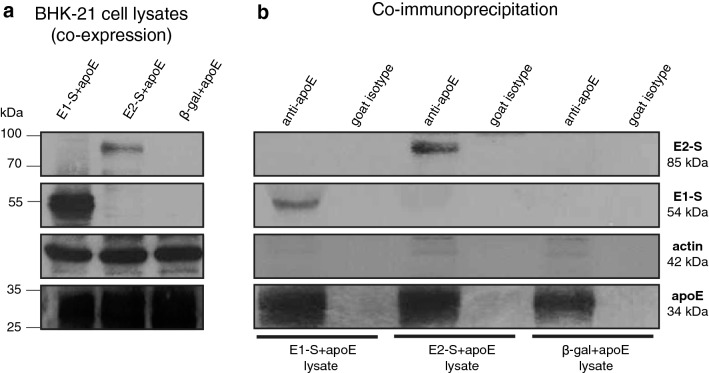
Evaluation of the interaction between apoE and HCV envelope glycoproteins fused to HBV S envelope protein (E1-S or E2-S) in co-immunoprecipitation assays. (**a**) Western-blot analysis of BHK-21 cell lysates co-expressing chimeric HBV–HCV E1-S or E2-S proteins, and apoE with the anti-E1 mAb (A4), anti-E2 mAb (H52), anti-apoE pAb (AB947) and anti-actin mAb (A1978). (**b**) Western-blot analysis of immune complexes from co-immunoprecipitation assays of BHK-21 cell lysates of interest with anti-apoE pAb (AB947) or with non-specific goat IgG (isotype control). Analyses were performed with the same antibodies as in panel (**a**).

The presence of apoE and chimeric E1-S or E2-S proteins in the baby hamster kidney-21 cell (BHK-21) lysates used for co-immunoprecipitation (co-IP) assays was evaluated by western blotting (Fig. 1a). The protein complexes co-immunoprecipitated and captured with anti-apoE antibodies coupled to G-sepharose beads were denatured and analyzed by the same technique. The proteins of interest were detected at the expected sizes, whereas no proteins were captured with a non-specific goat isotype (Fig. 1b). These results confirm the interactions between apoE and chimeric HBV–HCV proteins and the possibility of producing vaccine particles reproducing the interactions between these proteins.

### Efficient incorporation and presentation of apoE on the surface of the vaccine particles

**Figure 2:**
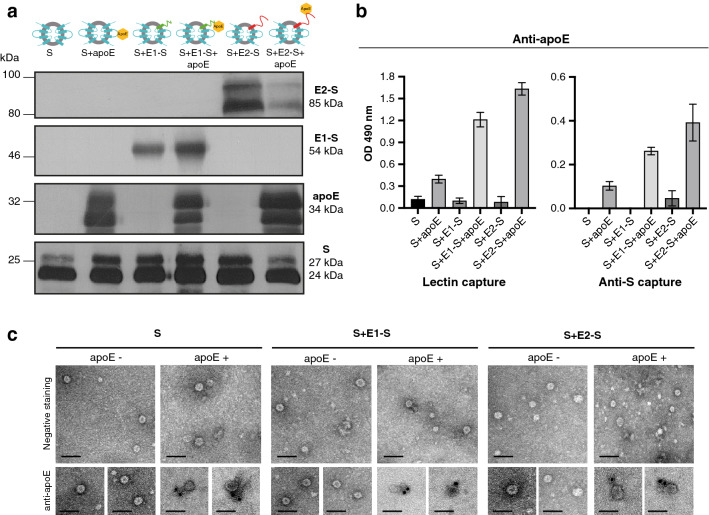
Characterization of the incorporation of apoE and its presentation in purified HBV–HCV subviral particles. (**a**) Western-blot analysis of subviral particles (75 µg/mL SHBsAg) and detection of proteins of interest with the anti-E1 mAb (A4), anti-E2 mAb (H52), anti-S pAb (70-HG15) and anti-apoE pAb (AB947). (**b**) Detection of apoE on the surface of particles by ELISA. Subviral particles (25 µg SHBsAg) were captured with lectin or an anti-S pAb (70-HG15), and apoE was detected with the anti-apoE (AB947) antibody. The experiment was performed once in triplicate, and the results are expressed as mean OD (490 nm) values ± SD. (**c**) Observation of subviral particles by TEM. Particles were analyzed by negative staining with 2% uranyl acetate solution (upper panels) and by the same method after immunogold staining with an anti-apoE pAb (AB947) (lower panels). Scale bars: 50 nm (in upper and lower panels).

SVPs produced by Chinese hamster ovary cell (CHO) clones stably expressing proteins of interest were purified with a mean purity of 13%. The incorporation of proteins of interest into purified SVPs was first verified by western blotting (Fig. 2a). We found that apoE was efficiently incorporated into SVPs, even those consisting of the wild-type (WT) HBV S protein alone. In particles containing the WT HBV S and E1-S or E2-S proteins, apoE clearly modulated the incorporation of chimeric HBV–HCV proteins. The amount of E1-S protein in apoE-containing SVPs was slightly higher than that in SVPs without apoE, whereas the opposite effect was observed for the incorporation of E2-S protein (Fig. 2a). Furthermore, the amount of apoE was always higher in particles bearing the E2-S protein than in those presenting the E1-S protein. These differences in incorporation were observed in several different purification batches. We subsequently checked that apoE was not only incorporated, but also exposed on the surface of the SVPs (Fig. 2b, c), by two different techniques. Enzyme-linked immunosorbent assays (ELISAs) (Fig. 2b), capturing particles with lectin or an anti-S antibody, showed that the amount of apoE exposed on the different particles followed the same trend, E2-S > E1-S > S, as observed in the western blotting analysis (Fig. 2a). Results obtained with particles captured with an anti-S antibody indicated that apoE is specifically bound to the SVPs. Finally, transmission electron microscopy (TEM) observations confirmed that apoE was present on the surface of SVPs and that particles had a similar morphology to that of WT HBV S particles (Fig. 2c).

### Differential incorporation and presentation of chimeric HBV–HCV envelope proteins in the presence and absence of apoE

**Figure 3:**
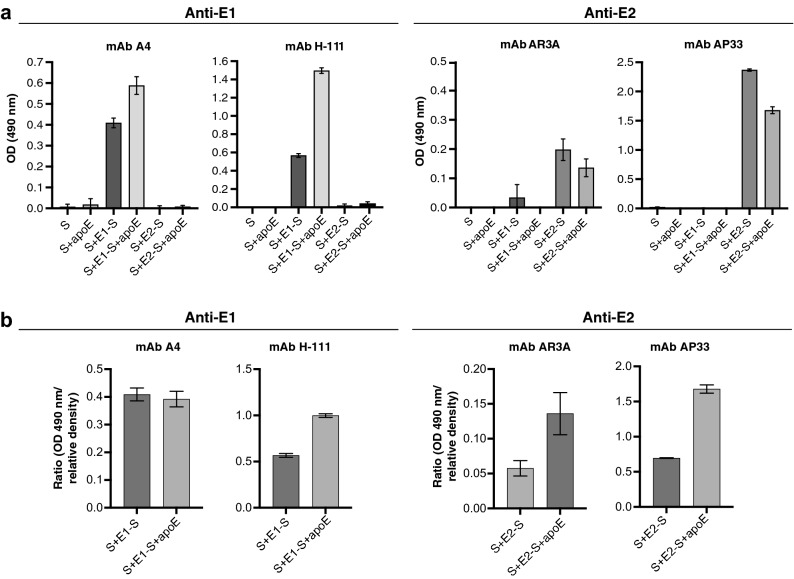
Analysis of the incorporation of E1-S and E2-S proteins and their presentation in purified HBV–HCV subviral particles**.** (**a**) Detection of HCV E1 or E2 proteins on the surface of SVPs by ELISA. Subviral particles (25 µg SHBsAg) were captured with an anti-S pAb (70-HG15), and proteins were detected on the surface of the particles with anti-E1 (mAbs A4 and H-111) or anti-E2 (mAbs AR3A and AP33) antibodies. Experiments were performed once in triplicate and results are expressed as the mean OD (490 nm) values ± SD. (**b**) The levels of chimeric HBV–HCV E1-S or E2-S proteins detected were normalized according to the amount of each protein incorporated into the subviral particles. The results are expressed here as the ratio of OD (490 nm) values obtained for the detection of chimeric HBV–HCV proteins by ELISA to the relative density of bands for the same proteins in subviral particles (Fig. 2a) ± SD.

We characterized this modulation of the incorporation of chimeric HBV–HCV envelope proteins into SVPs in more detail, by studying the HCV envelope proteins presented on the surface of particles in ELISAs with different antibodies (Fig. 3a). Both chimeric envelope proteins (E1-S and E2-S) are represented on the Supplementary Fig. S1 online, along with the epitopes corresponding to the antibodies used to detect those proteins. The detection of E1-S, by two monoclonal antibodies (mAbs) (A4 and H-111), revealed this protein to be present in larger amounts on S + E1-S + apoE particles than on S + E1-S particles. Conversely, the levels of E2-S protein detected with two mAbs (AP33 and AR3A) were poor when apoE was copresented on the SVPs. We analyzed the effect of apoE on the folding of chimeric E1-S and E2-S proteins, by normalizing ELISA results against the amount of these proteins incorporated into SVPs. The western blots were analyzed by densitometry (Fig. 2a), and the relative densities of E1-S and E2-S proteins were calculated in particles bearing apoE (S + E1-S + apoE and S + E2-S + apoE), in comparison to S + E1-S and S + E2-S, respectively. We found that there was 1.5 times more E1-S protein in apoE-containing particles than in S + E1-S SVPs, whereas the amount of E2-S protein in S + E2-S + apoE particles was lower, by a factor of 3.4, than that in S + E2-S particles. The optical density (OD)-to-relative density ratios (Fig. 3b) showed that higher levels of E1-S protein were detected on S + E1-S + apoE SVPs than on S + E1-S particles with the H-111 mAb, whereas this protein was detected at similar levels on both type of particle with the A4 mAb. The E2-S protein was much better recognized by the AP33 and AR3A mAbs (possessing a linear and a conformational epitope, respectively) when apoE was copresented on the SVPs. These results suggest that apoE improved the folding of both chimeric E1-S and E2-S proteins.

### Immunogenicity of vaccine particles with and without apoE

**Figure 4:**
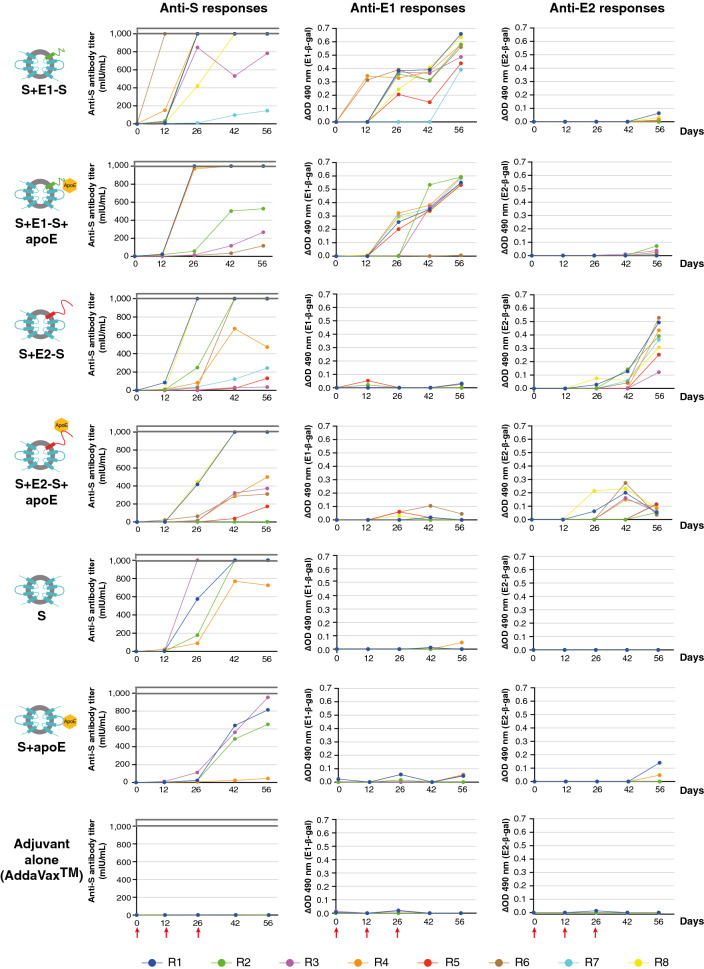
Humoral immune responses in rabbits immunized with HBV–HCV subviral particles with and without apoE presentation, with the AddaVax adjuvant. Specific anti-E1/anti-E2 and anti-S responses were evaluated in rabbit sera with “in-house” ELISAs and a routine immunoassay (ARCHITECT), respectively. Sera from rabbits immunized with S, S + apoE and AddaVax adjuvant alone were used as controls. Results for anti-E1/anti-E2 responses are expressed as the difference (Δ) in OD (490 nm) (anti-E1 or anti-E2—β-Gal), whereas anti-S titers are expressed in mIU/mL. Red arrows indicate the time at which immunizations occurred (days 0, 14 and 28).

Sera were collected from the immunized rabbits on days 0, 12, 26, 42 and 56, for the evaluation of humoral immune responses (Fig. 4). Anti-S responses were broadly similar for all groups of rabbits, but were slightly delayed in the groups immunized with apoE-bearing particles. The same phenomenon was observed when WT S vaccine particles were compared with S + apoE SVPs. The anti-E1 responses of rabbits immunized with S + E1-S + apoE SVPs tended to be slightly stronger, and remarkably more homogeneous (the kinetic curves of 7 of the 8 rabbits were similar) than those of rabbits immunized with S + E1-S particles. This observation can be explained by the observed improvements in E1-S protein folding when apoE was copresented, resulting in greater immunogenicity. The curves for the anti-E2 responses of rabbits immunized with S + E2-S + apoE SVPs showed peaks for anti-E2 antibody levels on day 42, followed by a rapid decrease in the response at day 56. These responses were clearly inferior on day 56 than those observed with S + E2-S particles, probably due to the much smaller amounts of E2-S protein observed in S + E2-S + apoE SVPs.

### Neutralization properties of the antibodies induced by immunization with vaccine particles with and without apoE

**Figure 5:**
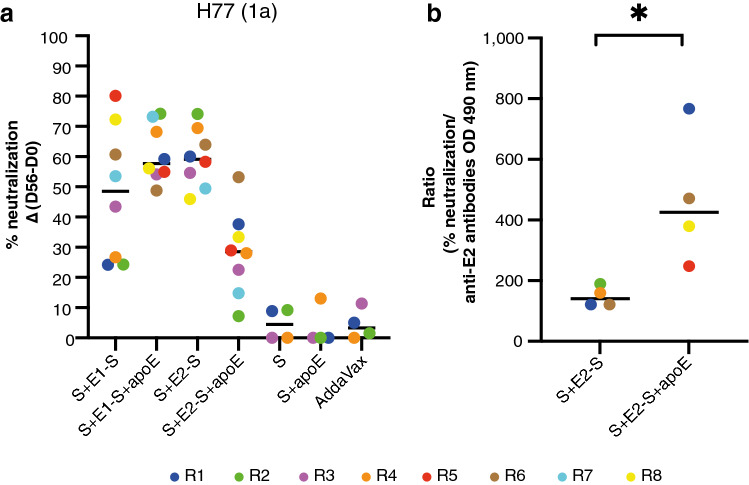
Neutralizing potential of antibodies induced by the immunization of rabbits with purified HBV–HCV subviral particles with and without apoE. (**a**) % neutralization of HCVcc genotype 1a. Rabbit serum samples (collected on days 0 and 56), at a 1:5 dilution, were incubated with HCVcc harboring HCV envelope proteins from genotype 1a (isolate H77), which were then used to infect Huh7.5 cells. Infection levels were determined 48 h post-infection, in a focus-forming unit staining assay. The % neutralization Δ(D56–D0) was determined by subtracting the % neutralization obtained with the pre-immune serum (day 0) from the one obtained with the post-immune serum (day 56) from the same rabbit. The assay was performed once in triplicate, and the results are expressed as mean values. Each colored point represents the % neutralization value calculated for a serum sample from an individual immunized rabbit, whereas the horizontal line represents the median value. The color code established for the antibody responses (Fig. 4), one color per rabbit, was conserved to simplify the correlation of results from both experiments. (**b**) Analysis of the neutralizing capacity of anti-E2 antibodies for the best four rabbits (% neutralization > the median) of each group (S + E2-S versus S + E2-S + apoE), related to the antibody levels, evaluated by ELISA on day 56. Results are expressed here as the ratio of the change in % neutralization between days 0 and 56 (Δ(D56–D0)) to the OD (490 nm) of anti-E2 antibodies obtained by ELISA on day 56 (Fig. 4). The groups were compared in non-parametric Mann–Whitney *U* test. (*) *p* = 0.0286.

Sera (collected on days 0 and 56) from rabbits immunized with SVPs containing or not containing apoE were evaluated in neutralization assays against hepatitis C virus generated in cell culture (HCVcc) harboring HCV envelope proteins from genotype 1a (isolate H77) (Fig. 5a). The percentage (%) neutralization achieved with serum from rabbits immunized with S + E1-S + apoE particles was higher than that achieved with serum from rabbits immunized with S + E1-S particles; the median and mean values for the S + E1-S + apoE group (57.68% and 61.09%) were higher than those for the S + E1-S group (48.53% and 48.17%). The data for the S + E1-S + apoE-immunized rabbits were more homogeneously distributed than those for the S + E1-S group (standard deviations of 9.53 and 22.10, respectively), confirming the phenomenon observed for the anti-E1 responses analyzed by ELISA (Fig. 4). Nevertheless, the % neutralization obtained following immunization with S + E2-S + apoE particles was lower (median 28.52% and mean 28.23%) than that achieved by immunization with S + E2-S SVPs (median 59.14% and mean 59.49%).

Given the much lower levels of anti-E2 antibodies detected on day 56 in rabbits immunized with S + E2-S + apoE particles (Fig. 4), and to optimize the evaluation of the neutralizing potential of these antibodies, we selected the best four rabbits and calculated the ratio of their % neutralization relative to the OD (490 nm) values obtained for anti-E2 responses by ELISA on day 56. The ratios (Fig. 5b) obtained for rabbits immunized with S + E2-S + apoE particles were higher than those for rabbits immunized with S + E2-S SVPs (median values of 342.2 and 154.7, respectively). The data for both groups were evaluated with the non-parametric Mann–Whitney *U* test and a significant difference (*p* value = 0.0286) was observed, indicating that the anti-E2 antibodies generated through the copresentation of apoE on chimeric HBV–HCV SVPs were of higher quality.

## Discussion

HCV-associated apoE has been shown to help the virus to avoid neutralization by antibodies isolated from chronically infected patients. Functional analyses with human mAbs showed that conformational epitopes of E2 protein were exposed after apoE depletion and that the level and conformation of virion-associated apoE affected the ability of the virus to escape neutralization by antibodies^29^. These important findings revealed a novel strategy contributing to ability of HCV to escape the immune system and establish chronic infection. We hypothesized that immunogens mimicking epitopes at the interface between HCV envelope proteins and apoE might generate a better neutralizing humoral immune response against HCV. The objective of this study was, therefore, to test this hypothesis using our bivalent HBV–HCV vaccine model.

We first showed, through co-IP experiments, that chimeric E1-S and E2-S proteins were able to interact with apoE, even in the context of fusion with the HBV S protein (Fig. 1). The interaction between apoE and the E1–E2 heterodimer is well documented^30–32^, but conflicting results have been obtained in previous studies, with one study showing that the E1 protein was responsible for this interaction^30^, whereas two other studies implicated the E2 protein^31,32^. We found that both our chimeric HBV–HCV envelope proteins were able to interact with apoE, making it possible to investigate the incorporation of apoE into vaccine particles. We successfully produced HBV–HCV SVPs bearing apoE, the presence of which was confirmed by western blotting, ELISA and TEM experiments (Fig. 2). Curiously, apoE was also incorporated into particles containing only the WT HBV S protein, suggesting a direct interaction between these two proteins. This interaction was confirmed by co-IP experiment between apoE and the HBV S protein (see Supplementary Fig. S2a online; procedures are also described in the Supplementary Information file). Indeed, this result is consistent with a recent study reporting that an association of apoE with HBV is essential for virus production^34^. Nevertheless, this interaction raises the question of the protein domains involved in the association between apoE and the chimeric proteins. ELISA and western blotting experiments showed that larger amounts of apoE were incorporated into particles containing E1-S or E2-S proteins than into particles containing only the WT HBV S protein, suggesting a possible cumulative effect of these different interactions. To verify this hypothesis, we were able to demonstrate by co-IP experiments that native HCV envelope proteins (both E1 and E2) interact with apoE (see Supplementary Fig. S2b online; procedures are also described in the Supplementary Information file). Moreover, the vaccine particles bearing the chimeric E2-S protein incorporated the largest amounts of apoE, implying either stronger protein–protein interactions or an involvement of more than one protein domain in this interaction. To verify that this difference was not due to a problem in protein expression, we analyzed the CHO cell clone lysates through western blotting during the production of vaccine particles (see Supplementary Fig. S3 online; procedures are also described in the Supplementary Information file). We observed that the amount of apoE happened to be similar in lysates from CHO-S + E1-S + apoE and CHO-S + E2-S + apoE clones. Therefore, this may reflect the reported higher stringency of the apoE-E2 interaction than of the apoE-E1 interaction^32^. In any case, despite the large amount of apoE incorporated into SVPs, particularly those carrying the E2 protein, TEM analyses showed that the particles generated had the same morphology as the original HBV vaccine.

We found that the incorporation of E1-S was not affected by the presence of apoE, whereas that of E2-S was strongly impaired in the presence of apoE. The reasons for this difference in incorporation are unknown, but may be related to the amount of apoE detected in these particles. This phenomenon was repeatedly observed in the preparation of several batches of purified SVPs. Indeed, these results are consistent with previous studies showing that, in a viral context, apoE is exposed at a higher copy number than the HCV E2 protein^35,36^. It is, therefore, possible that, during the SVP budding process, a strong interaction occurs between E2-S and apoE, or that the presence of a large number of apoE molecules interferes with the incorporation of the chimeric E2-S protein into particles, instead favoring the integration of WT S proteins. In any case, more experiments are required to validate these hypotheses and to shed light on the process of particle formation.

Following normalization of the detection rates of chimeric E1-S and E2-S proteins on the surface of the SVPs, we found that apoE improved the folding of these proteins. We used mAbs directed against HCV envelope E1 and E2 proteins for the recognition of chimeric envelope proteins (Fig. 3). The AR3A mAb was able to detect its conformational epitope in the HCV E2 protein, and this detection was improved by the incorporation of apoE into particles. The AP33 mAb, the epitope of which is located in the conserved region downstream (aa 412–423) from hypervariable region 1 of the E2 protein, also recognized the E2 protein more efficiently when apoE was present. Similar findings were obtained for the H-111 mAb, one of the few antibodies with neutralizing activity directed against the HCV E1 protein. The access of antibodies to these epitopes has already been described as a presumably important element in the development of an effective HCV vaccine^37^. Our findings therefore show that the HBV–HCV particles are a potentially good model for mimicking the apoE/HCV envelope-glycoprotein interface.

An analysis of the immune responses of rabbits vaccinated with SVPs showed that apoE had no statistically significant impact on the level of anti-S antibodies by day 56. However, anti-S responses were delayed in rabbits immunized with vaccine particles bearing apoE, possibly due to the immunomodulatory functions attributed to apoE^38^. Anti-E1 antibody responses were efficient with and without apoE, but were much more homogeneous for rabbits immunized with S + E1-S + apoE SVPs (Fig. 4). A similar homogeneity was found for the % neutralization of HCVcc genotype 1a, with slightly higher median values for rabbits immunized with S + E1-S particles (Fig. 5). We cannot rule out the possibility that this improvement in neutralization was due to the induction of anti-apoE antibodies in immunized animals. Unfortunately, we were unable to establish an ELISA for the specific detection of such antibodies. Furthermore, this increase in % neutralization did not seem to be related to a neutralizing activity of potential anti-apoE antibodies, because animals immunized with S + apoE particles did not develop a neutralizing response against HCV. The results for the E1 protein are promising, as S + E1-S + apoE SVPs could be used in a mixture of vaccine particles. In a previous study, we evaluated this strategy with particles bearing the E1 and E2 proteins separately and found that anti-E1 and anti-E2 antibodies had additive neutralizing properties, suggesting that this approach would be relevant for inducing dual anti-E1 and anti-E2 responses^26^.

Unfortunately, the anti-E2 responses obtained in rabbits immunized with S + E2-S + apoE particles were rather disappointing when compared with those obtained for rabbits immunized with S + E2-S SVPs. However, these results are not surprising, as only very small amounts of E2-S protein were incorporated into the apoE-containing particles. The anti-E2 response displayed a small peak at day 42 (above the anti-E2 responses from the S + E2-S group at that time point), before drastically falling by day 56, whereas antibody levels continued to increase in animals vaccinated with S + E2-S SVPs (Fig. 4). This phenomenon remains unexplained and requires further investigation. These results may be related to the complex roles of apoE in modulating the immune response^38^. We retrospectively performed neutralization assays of HCVcc (genotypes 1a and 2a) using serum samples collected at day 42 from rabbits immunized with S + E2-S + apoE or S + E2-S particles (see Supplementary Fig. S4 online). The % neutralization obtained with sera from rabbits immunized with apoE-containing particles were slightly higher than those obtained with sera from the S + E2-S-immunized group, which was expected due to the somewhat superior antibody titers. In contrast, by day 56, the neutralizing responses in animals immunized with S + E2-S + apoE particles were disappointing, as for the anti-E2 antibody levels determined by ELISA, with a much lower % neutralization than for immunization with S + E2-S particles. Nevertheless, after the normalization of % neutralization based on antibody levels at day 56, the antibodies generated in the presence of apoE were found to have better neutralizing properties than those induced by S + E2-S SVPs (Fig. 5). To evaluate the neutralizing potential of those antibodies against another HCV genotype, we performed neutralization assays of HCVcc genotype 2a (isolate JFH1) and we observed comparable results to the homologous neutralization (see Supplementary Fig. S5 online). Overall, the antibodies induced by our vaccines maintained their neutralizing properties at high serum dilutions (see Supplementary Fig. S6 online). These results suggest that this approach is potentially interesting for vaccine strategies aiming to increase the neutralizing potential of anti-E2 antibodies. In the case of our bivalent HBV–HCV vaccine model, the problem of the low level of E2-S protein incorporation into apoE-containing particles will need to be resolved. We therefore now plan to modulate the expression of apoE in order to decrease the apoE/E2 ratio in the secreted SVPs.

In conclusion, this study shows that both chimeric HBV–HCV proteins were able to interact with apoE. The presentation of E1-S or E2-S proteins, in complex with apoE, resulted in the induction of a more efficient HCV-neutralizing humoral response than the presentation of these proteins without apoE. The results obtained with our HBV–HCV vaccine model could potentially be exploited for immunization with E1-containing SVPs. Unfortunately, apoE greatly decreased the incorporation of E2 protein into the vaccine particles. This problem will need to be addressed for the development of a similar strategy for immunization with particles bearing the E2 protein. In spite of that, these results demonstrate the potential benefits of incorporating apoE into vaccine particles for the presentation of HCV envelope proteins to the immune system, and this protein may be useful for other envelope-based vaccine approaches against HCV.

## Materials and methods

The authors of this article certify that all experiments were performed in accordance with relevant guidelines and regulations, including the recommendations of the ARRIVE guideline.

### Analysis of interactions between apoE and chimeric HBV–HCV envelope proteins through co-immunoprecipitation assays

Co-IP assays were performed on lysates of BHK-21 cells co-expressing apoE and chimeric HBV–HCV envelope proteins (E1-S or E2-S) (HBV subtype *adw* and HCV genotype 1a) or β-galactosidase (β-gal) from original Semliki Forest virus (SFV)-derived vectors. We ensured that all these proteins of interest were produced by cotransfecting cells with each of the previously described pSFV-E1-S, pSFV-E2-S and pSFV3-β-gal vectors^23^ separately, and with a new pSFV1-apoE vector (β-gal + apoE, E1-S + apoE or E2-S + apoE). This new plasmid was generated by the same strategy as the other SFV-derived vectors^23^. Briefly, the gene encoding human apoE isoform E3 was amplified by polymerase chain reaction (PCR) from the pOTB7-apoE plasmid (kindly provided by Dr. Catherine Schuster)^39^, with the Phusion high-fidelity DNA polymerase (New England Biolabs) and primers flanked by *Bam*HI restriction sites (underlined), forward: 5′-ATAGGATCCATGAAGGTTCTGTGGGCTGC-3′ and reverse: 5′-ATAGGATCCTTAGTGATTGTCGCTGGGCACAG-3′. The PCR product was inserted into the pGEM^®^-T vector (pGEM-T Easy Vector System; Promega), from which it was excised with *Bam*HI enzyme (New England Biolabs) for insertion into the *Bam*HI restriction site of the pSFV1 vector (Invitrogen). All PCR products were verified by DNA sequencing. Recombinant SFV RNA synthesis, and the cotransfection and lysis of BHK-21 cells were performed as described elsewhere^24^.

For co-IP assays, the previously described cell lysates and either goat polyclonal (pAb) anti-apoE antibody (AB947; Millipore; AB_2258475) or goat-isotype control (R&D Systems; AB_354267) were incubated overnight at 4 °C with rec-Protein G-Sepharose 4B conjugate beads (Invitrogen) in 1X phosphate-buffered saline (PBS; Gibco) solution. The beads were washed in 0.1% Triton X-100 (Sigma-Aldrich) in 1X PBS solution, and the immune complexes were analyzed by western blotting in denaturing conditions. For this purpose, samples containing equal amounts of total protein were prepared in reducing sample buffer (1X Laemmli buffer supplemented with 25% β-mercaptoethanol (Sigma-Aldrich)) and boiled for 5 min. They were then subjected to sodium-dodecyl sulfate–polyacrylamide gel electrophoresis (SDS-PAGE) in 12% acrylamide gels and the resulting bands were electroblotted onto a nitrocellulose membrane (Amersham Protran; GE Healthcare). Non-specific sites were blocked by incubation with 2% non-fat dry milk in 1X Tris-buffered saline (TBS; Euromedex) solution for 1 h at room temperature. Membranes were incubated overnight at 4 °C with a mouse mAb directed against E1 (clone A4; generously provided by Dr. Harry Greenberg)^40^, a mouse anti-E2 mAb (clone H52; generously provided by Dr. Jean Dubuisson)^41^, an anti-apoE pAb (AB947) or a mouse anti-actin mAb (A1978, clone AC-15; Sigma-Aldrich; AB_476692). The membranes were then washed with 0.3% Tween (Sigma-Aldrich) in 1X TBS solution and incubated with horseradish peroxidase-conjugated secondary antibodies for 1 h at room temperature. Membranes were washed again with 0.3% Tween buffer and then developed by enhanced chemiluminescence. BHK-21 cell lysates, used for co-IP assays, were also analyzed according to the same western blotting protocol.

### Development of stable CHO clones co-expressing apoE and chimeric HBV–HCV envelope proteins

Stable clones of CHO cells co-expressing WT HBV S protein (HBV subtype *adw*) and chimeric HBV–HCV E1-S or E2-S envelope proteins (HCV genotype 1a) were generated by lentiviral transduction, as described elsewhere^23^. New clones co-expressing these proteins of interest and apoE were developed according to the same strategy. Briefly, CHO-S, CHO-S + E1-S and CHO-S + E2-S clones were stably transduced with a lentiviral expression vector encoding apoE and the blasticidin-S deaminase. Clones (CHO-S + apoE, CHO-S + E1-S + apoE and CHO-S + E2-S + apoE) resistant to the antibiotics hygromycin and blasticidin (InvivoGen) were amplified and protein levels were evaluated by western blotting, as described above, with anti-E1 mAb (A4), anti-E2 mAb (H52), anti-apoE pAb (AB947) and goat anti-S pAb (70-HG15; Fitzgerald; AB_231585).

### Production and purification of vaccine particles

CHO clones (CHO-S, CHO-S + apoE, CHO-S + E1-S, CHO-S + E1-S + apoE, CHO-S + E2-S and CHO-S + E2-S + apoE) were cultured in DMEM/F12 medium (Gibco) supplemented with 10% heat-inactivated fetal calf serum (FCS; Eurobio Scientific), 100 U/mL penicillin and 100 µg/mL streptomycin (penicillin–streptomycin; Gibco) at 37 °C, under a humidified atmosphere containing 5% CO_2_. Clones were gradually adapted to a low-serum (2.5% FCS) Advanced DMEM/F12 medium (Gibco) appropriate for mass production. In some cases, depending on the clone of interest, the medium was supplemented with antibiotics (hygromycin and/or blasticidin). Each clone was cultured in HYPERFlask cell culture vessels (Corning). Every 10–15 days, the supernatant was harvested and the culture vessels were fed with cells and fresh media.

SVPs (S, S + apoE, S + E1-S, S + E1-S + apoE, S + E2-S and S + E2-S + apoE) were purified from the supernatants of cell cultures as described elsewhere^23,24^. Briefly, total proteins were precipitated by incubation overnight at 4 °C with ammonium sulfate ((NH_4_)_2_SO_4_). The precipitated proteins were recovered by centrifugation and dialyzed against Tris-NaCl-EDTA (TNE) buffer (10 mM Tris–HCl [pH 7.5]/100 mM NaCl/1 mM EDTA) overnight at 4 °C. The SVPs were purified by successive isopycnic ultracentrifugations on cesium chloride (CsCl) gradients. SHBsAg-positive fractions of gradients were identified in a previously described “in-house” ELISA^23^, pooled and dialyzed against TNE buffer overnight at 4 °C. Purified vaccine particles were concentrated with the Amicon Ultra-15 100 k device (Millipore) and their final concentrations of SHBsAg were determined with the same “in-house” ELISA, with a sequentially diluted recombinant hepatitis B surface antigen (HBsAg) subtype *adw* (R86872; Meridian Life Science) as the standard. SVP purity was calculated as the ratio of SHBsAg concentration to total protein concentration assessed by determining the absorbance measured at 280 nm with a NanoDrop spectrophotometer (DeNovix).

### Analysis of protein incorporation into purified SVPs

The presence of proteins of interest (S, chimeric E1-S or E2-S proteins, and apoE) in purified SVPs was first evaluated by western blotting (with an equal concentration of 75 µg/mL SHBsAg), as described above, with the anti-E1 mAb (A4), anti-E2 mAb (H52), anti-apoE pAb (AB947) and anti-S pAb (70-HG15). Densitometry with ImageJ software (NIH) was performed to measure the intensity of the E1-S and E2-S bands on western blots (Fig. 2a). The relative densities of these protein bands were calculated in pairs (S + E1-S and S + E1-S + apoE; S + E2-S and S + E2-S + apoE) with the particles having the lowest intensities for the E1-S or E2-S bands used as the reference (relative density = 1).

We evaluated the presence of apoE on the surface of particles by ELISA and TEM. For ELISA, SVPs (containing 25 µg SHBsAg) were captured by lectin (Sigma-Aldrich) or an anti-S pAb (70-HG15) in a 96-well plate (Immulon 2 HB; Thermo Fisher Scientific), and apoE was detected with a specific antibody (AB947). The assay was performed once in triplicate, and results are expressed as mean OD (490 nm) ± standard deviation (SD). For TEM, formvar/carbon-coated nickel grids were placed on drops of samples for five minutes, and were then rinsed twice with 1X PBS. Then, grids were incubated on a drop of 1% bovine serum albumin (Sigma-Aldrich)-PBS, and then on a drop of goat anti-apoE pAb (AB947) diluted 1:100 in 1X PBS, for one hour. The grids were washed six times with 1X PBS (5 min each), and then incubated for 1 h on a drop of gold-conjugated (6 nm) donkey-anti-goat IgG (Aurion) diluted 1:30 in 1X PBS. Grids were washed again with 6 drops of 1X PBS, post-fixed in 1% glutaraldehyde and rinsed with three drops of distilled water. Negative staining was then performed with three consecutive contrast steps, using 2% uranyl acetate (Agar Scientific), for analysis under a transmission electron microscope (JEOL 1011). Negative staining of the samples without labeling was also performed.

The incorporation and folding of chimeric E1-S and E2-S proteins at the surface of the purified SVPs were also evaluated by an ELISA based on the capture of SVPs (25 µg SHBsAg) in 96-well plates (Nunc Immuno plates; Thermo Fisher Scientific) with the anti-S pAb (70-HG15), followed by detection with the anti-E1 mAb (A4), the human anti-E1 mAb (clone H-111; generously provided by Dr. Steven Foung)^42^, the human anti-E2 mAb (AR3A; kindly provided by Dr. Mansun Law)^43^ or the mouse anti-E2 mAb (clone AP33; Genentech)^44^. The assay was performed once in triplicate, and the results are expressed as mean OD values (490 nm) ± SD. Protein levels were normalized by calculating the ratios between the ODs (490 nm) obtained for each anti-E1 and anti-E2 antibody and the relative density of the E1-S and E2-S proteins, respectively, calculated as described above.

### Immunization of female New Zealand rabbits

Vaccine doses were prepared with 15 µg of purified SVPs mixed in a 1:1 ratio (vol:vol) with AddaVax (InvivoGen), a squalene-based oil-in-water nanoemulsion. Female New Zealand rabbits were immunized by Agro-Bio (La Ferté Saint-Aubin, France), a company accredited by the French authorities for the performance of protocols on laboratory animals in accordance with European Directive 2010/63/EU. The protocol was approved by Agro-Bio Ethics Committee C2EA-99 (affiliated to the National Committee of Ethics on Animal Experimentation of the French Ministry of Research, agreement B45-146-01).

Groups of eight rabbits were immunized subcutaneously with particles of interest (S + E1-S, S + E1-S + apoE, S + E2-S or S + E2-S + apoE), and control groups of four rabbits were injected with AddaVax adjuvant alone, or with S or with S + apoE particles on days 0, 14 and 28. Serum samples were collected from rabbits at various time points (days 0, 12, 26, 42 and 56) for the characterization of humoral responses.

### Analysis of anti-S, anti-E1 and anti-E2 responses

Anti-S antibodies were quantified with the ARCHITECT system (Abbott Laboratories). Anti-E1 and anti-E2 responses were evaluated with an “in-house” ELISA based on the use of lysates of BHK-21 cells expressing HCV E1 or E2 proteins, versus β-gal (control), from SFV-derived vectors. Cell lysates containing β-gal, HCV E1 or E2 proteins were obtained as described elsewhere^24,26^. A detailed description of ELISAs can be found in the Supplementary Information file. The results for each sample are expressed as the difference between the mean OD (490 nm) for HCV E1 or E2 wells and the mean of OD (490 nm) for β-gal wells.

### Neutralization of HCV in cell culture

The neutralizing properties of antibodies were evaluated in vitro with the HCVcc model. First, HCVcc harboring HCV envelope proteins from genotype 1a (isolate H77)^45^ were generated by the transfection of hepatoma Huh7.5 cells (kindly provided by Prof. Charles Rice)^46^ with the full genomic HCV RNA, transcribed in vitro, as previously described^47^. Viral infectivity was determined in a focus-forming units (FFU) staining assay described elsewhere^24^. In addition, we verified the presence of apoE in HCVcc used for neutralization assays (data not shown).

In neutralization assays, 50 µL of inoculum, corresponding to 100 FFUs, was mixed with 25 µL of a 1:5 dilution of rabbit serum (day 0 or 56) and incubated for 1 h at 37 °C. We then infected 10,000 Huh7.5 cells with 75 µL of the virus-serum mixture for 6 h at 37 °C, under a humidified atmosphere containing 5% CO_2_. The inoculum was then removed and replaced with fresh medium and the cells were incubated for a further 37 °C. Infectivity was evaluated 48 h post-infection, as described above. The % neutralization Δ(D56–D0) was determined by subtracting the % neutralization obtained with the pre-immune serum (day 0) from the one obtained with the post-immune serum (day 56) from the same rabbit. The assay was performed once in triplicate, and the results are expressed as mean % neutralization values for each rabbit.

The neutralizing potential of the antibodies from the best four rabbits (% neutralization > median value) from the S + E2-S and S + E2-S + apoE groups was analyzed by calculating the ratio of % neutralization to the corresponding anti-E2 responses (OD 490 nm) at day 56. Results are expressed as ratios and the median value is shown.

### Statistical analysis

Data analyses were conducted with Prism8 (GraphPad), and non-parametric Mann–Whitney *U* tests for group comparisons; *p* values < 0.05 were considered significant.

## Supplementary Information

Supplementary Figure 1.

Supplementary Figure 2.

Supplementary Figure 3.

Supplementary Figure 4.

Supplementary Figure 5.

Supplementary Figure 6.

Supplementary Information.

## Data Availability

The datasets generated during the current study are available from the corresponding author on reasonable request.

## Abbreviations

apoE: Apolipoprotein E
β-gal: Beta-galactosidase
BHK-21: Baby hamster kidney-21 cells
CHO: Chinese hamster ovary cells
co-IP: Co-immunoprecipitation
DAA: Direct-acting antiviral
E1: HCV envelope glycoprotein 1
E1-S: Chimeric HBV–HCV E1 protein
E2: HCV envelope glycoprotein 2
E2-S: Chimeric HBV–HCV E2 protein
ELISA: Enzyme-linked immunosorbent assay
FCS: Fetal calf serum
FFU: Focus-forming unit
HBsAg: Hepatitis B surface antigen
HBV: Hepatitis B virus
HCC: Hepatocellular carcinoma
HCV: Hepatitis C virus
HCVcc: Hepatitis C virus in cell culture
LVP: Lipoviral particle
mAb: Monoclonal antibody
Nabs: Neutralizing antibodies
OD: Optical density
pAb: Polyclonal antibody
PBS: Phosphate-buffered saline
PCR: Polymerase chain reaction
S: HBV small envelope protein
SHBsAg: Small hepatitis B surface antigen
SD: Standard deviation
SDS-PAGE: Sodium dodecyl sulfate–polyacrylamide gel electrophoresis
SFV: Semliki Forest virus
SVP: Subviral particle
TBS: Tris-buffered saline
TEM: Transmission electron microscopy
TNE: Tris-NaCl-EDTA
WHO: World Health Organization
WT: Wild-type
