# Supplementary figures and images for: Incorporation of apolipoprotein E into HBV–HCV subviral envelope particles to improve the hepatitis vaccine strategy
Source: Sci Rep. 2021 Nov 8;11:21856. doi: 10.1038/s41598-021-01428-7 (PMC8575973; doi:10.1038/s41598-021-01428-7)

E1-S

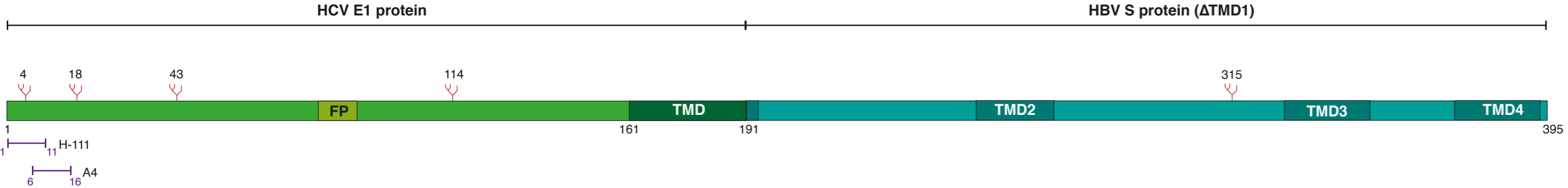

E2-S

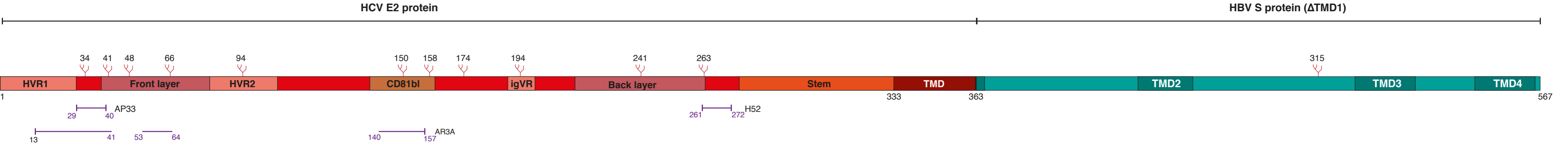

Supplement: Supplementary file 1 — Supplementary Figure 1. [file 41598_2021_1428_MOESM1_ESM.pdf]

**a**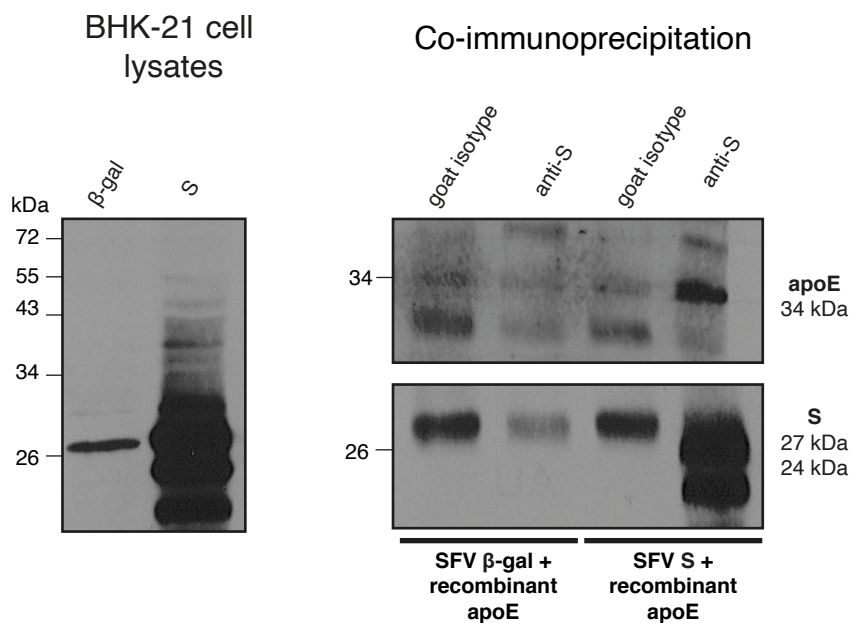**b**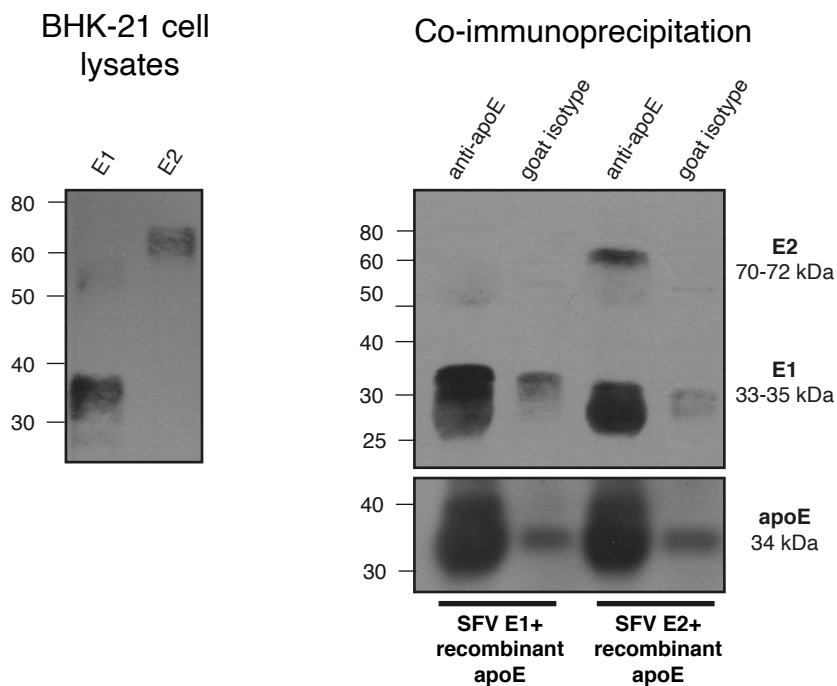

Supplement: Supplementary file 2 — Supplementary Figure 2. [file 41598_2021_1428_MOESM2_ESM.pdf]

## CHO cell lysates

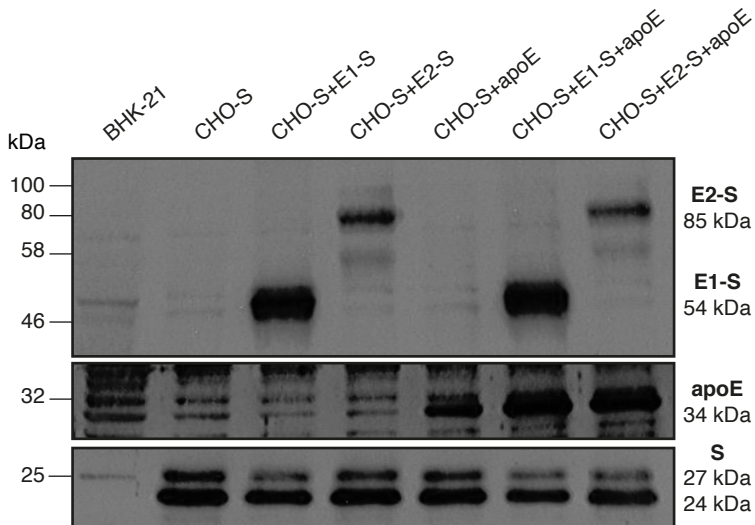

Supplement: Supplementary file 3 — Supplementary Figure 3. [file 41598_2021_1428_MOESM3_ESM.pdf]

HCVcc genotype 1a  
(H77/JFH1)

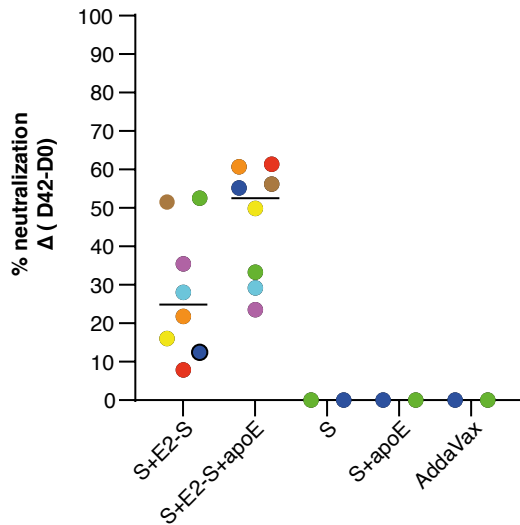

HCVcc genotype 2a  
(JFH1)

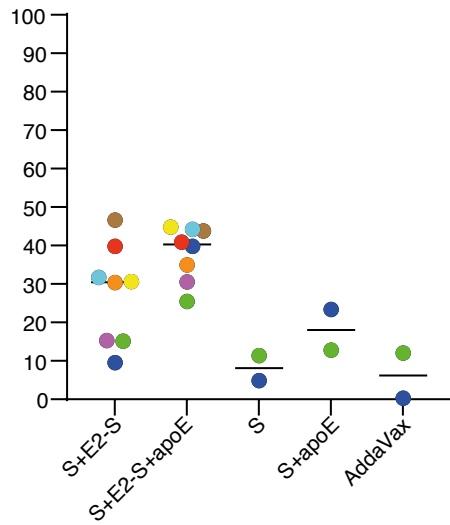

● R1 ● R2 ● R3 ● R4 ● R5 ● R6 ● R7 ● R8

Supplement: Supplementary file 4 — Supplementary Figure 4. [file 41598_2021_1428_MOESM4_ESM.pdf]

**a**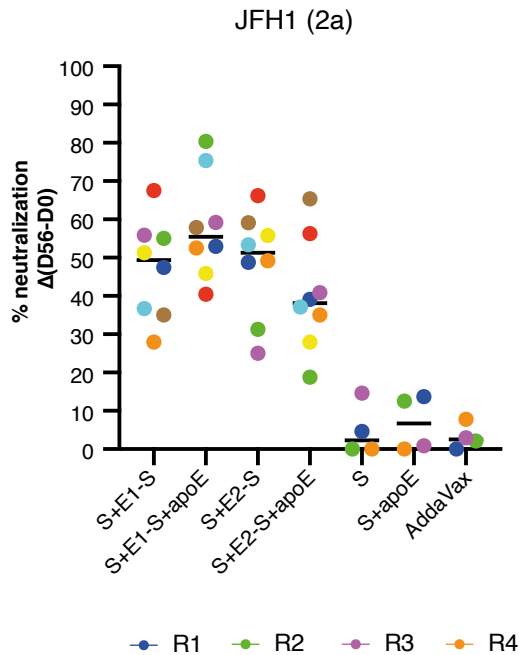**b**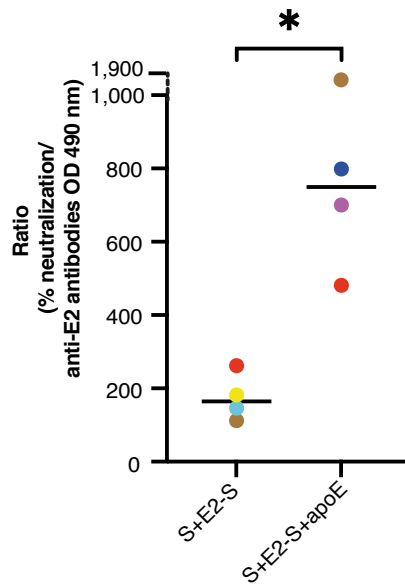

Supplement: Supplementary file 5 — Supplementary Figure 5. [file 41598_2021_1428_MOESM5_ESM.pdf]

## HCVcc genotype 1a (H77/JFH1)

## HCVcc genotype 2a (JFH1)

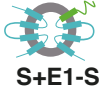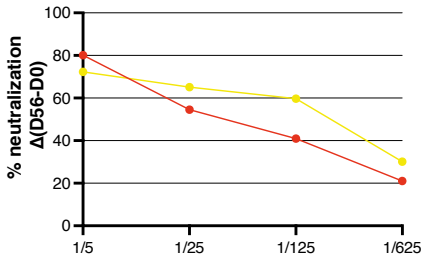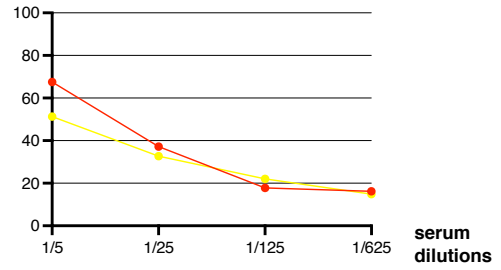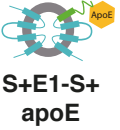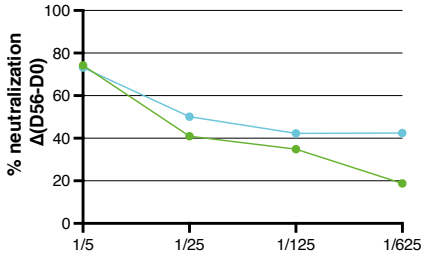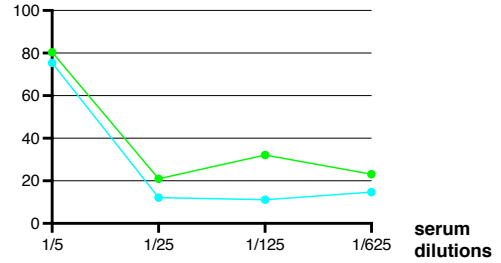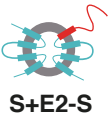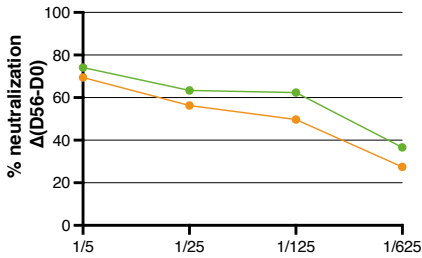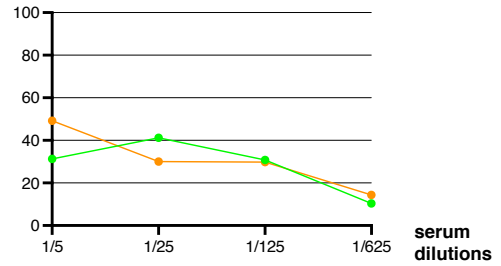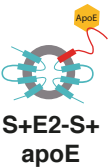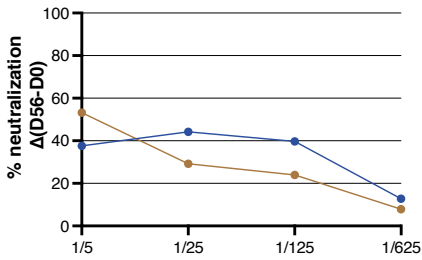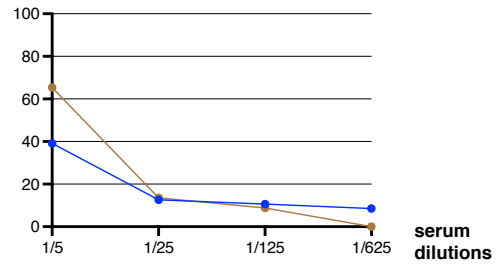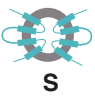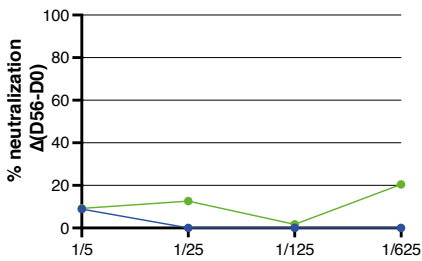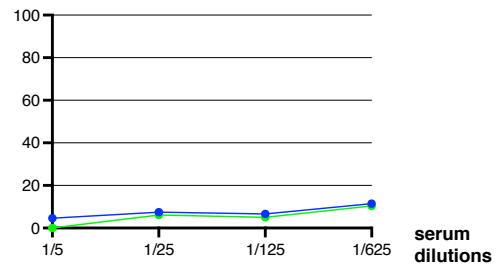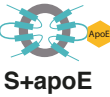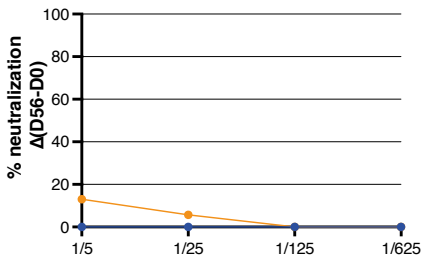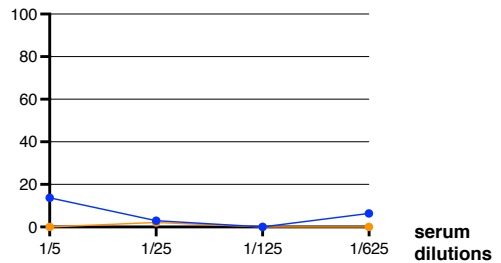

**Adjuvant  
alone  
(AddaVax™)**

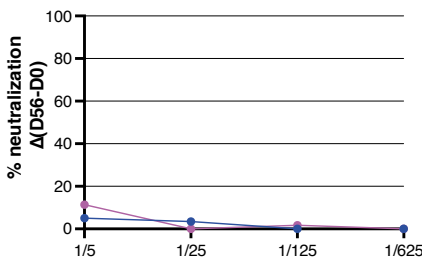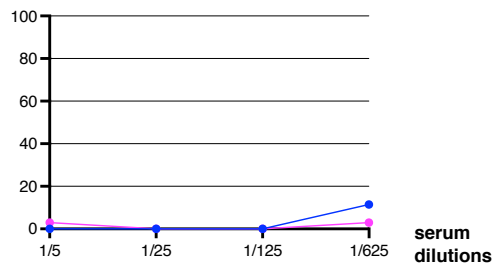

● R1 ● R2 ● R3 ● R4 ● R5 ● R6 ● R7 ● R8

Supplement: Supplementary file 6 — Supplementary Figure 6. [file 41598_2021_1428_MOESM6_ESM.pdf]
