# Supplementary material for: Incorporation of apolipoprotein E into HBV–HCV subviral envelope particles to improve the hepatitis vaccine strategy
Source: Sci Rep. 2021 Nov 8;11:21856. doi: 10.1038/s41598-021-01428-7 (PMC8575973; doi:10.1038/s41598-021-01428-7)
Supplement: Supplementary file 7 — Supplementary Information. [file 41598_2021_1428_MOESM7_ESM.docx]

**Incorporation of apolipoprotein E into HBV-HCV subviral envelope particles to improve the hepatitis vaccine strategy**

Elsa Gomez-Escobar,^1^ Julien Burlaud-Gaillard,^1,2^ Clara Visdeloup,^1^ Adeline Ribeiro E Silva,^1^ Pauline Coutant,^1^ Philippe Roingeard,*^✝1, 2^ Elodie Beaumont*^✝1^

^1^ Inserm U1259 MAVIVH, Université de Tours and CHRU de Tours, Tours, France

^2^ Plate-Forme IBiSA des Microscopies, PPF ASB, Université de Tours and CHRU de Tours, Tours, France

^✝^ These authors jointly supervised this work.

^*^Corresponding authors

# SUPPLEMENTARY MATERIALS AND METHODS

**Analysis of anti-E1 and anti-E2 responses**

ELISAs were performed in 96-well Immulon 2 HB plates coated by overnight incubation with 2 µg/well lectin from *Galanthus nivalis* (Sigma-Aldrich) at 4°C. Non-specific binding sites were saturated by incubation with a blocking solution (10% non‑fat dry milk + 5% sheep serum-TBS 1X) for 4 hours at 37°C. We then added 2 µg/well cell lysate (ß‑galactosidase (ß‑gal), HCV E1 or E2 proteins) and incubated overnight at 4°C. A second blocking of non‑specific binding sites was then performed, as described above. We added 100 µL of serum from immunized rabbits (day 0, 12, 26, 42 or 56) to the plates and incubated for 1 hour at 37°C. Secondary HRP‑conjugated goat F(ab')_2_ anti-rabbit IgG (H+L) antibody (Southern Biotech) was added and the plates were incubated for 1 hour at 37°C. We then added O‑phenylenediamine dihydrochloride (SIGMAFAST™ OPD; Sigma-Aldrich) as an HRP substrate. The reaction was stopped after 15 minutes by adding 2 N H_2_SO_4_, and absorbance was read at 490 nm.

**Analysis of the expression levels of proteins of interest of CHO clones through western blotting**

We lysed CHO clones stably producing proteins of interest (S and E1-S, E2-S or apoE) in 1% Triton X-100 in 1X PBS buffer supplemented with protease inhibitors. Cell lysates were analyzed by western blotting in denaturing conditions (as described in the main manuscript) with goat anti-S (70-HG15) and anti-apoE (AB947) polyclonal antibodies (pAbs), and mouse anti-E1 (A4) and anti-E2 (H52) monoclonal antibodies (mAbs).

**Analysis of interactions between apoE and HBV S protein or HCV envelope proteins through co-immunoprecipitation assays**

Co-immunoprecipitation (co-IP) assays were performed on lysates of BHK-21 expressing β-gal, HBV S protein (*adw* subtype) or HCV envelope proteins (E1 or E2) (genotype 1a) from original Semliki Forest virus (SFV)-derived vectors. First, we ensured that proteins of interest were produced by transfecting cells with each of the previously described pSFV3-β-gal, pSFV-SHBs^adw^, pSFV-E1 and pSFV-E2 plasmids,^1,2^ separately. Recombinant SFV RNA synthesis, and the transfection and lysis of BHK-21 cells were performed as described elsewhere.^3^

For co-IP assays, the previously described cell lysates were pre-incubated with a purified native human apoE (isoform E3; My BioSource). Then, the protein complexes, with either an anti-apoE pAb (AB947), an anti-S pAb (70-HG15) or a goat-isotype control (R&D Systems), were incubated overnight at 4°C with rec-Protein G-Sepharose^®^ 4B conjugate beads in 1X PBS. The beads were washed in 0.1% Triton X-100 in 1X PBS buffer, and the immune complexes were analyzed by western blotting in denaturing conditions (as described in the main manuscript) with anti-E1/anti-E2 mAbs (A4 and H52) or anti-S (70-HG15), and anti-apoE (AB947) pAbs. BHK-21 cell lysates, used for co-IP assays, were also analyzed according to the same western blotting protocol.

**REFERENCES**

1. Patient, R., Hourioux, C., Vaudin, P., Pagès, J.-C. & Roingeard, P. Chimeric hepatitis B and C viruses envelope proteins can form subviral particles: implications for the design of new vaccine strategies. *N. Biotechnol.* **25**, 226–234 (2009).

2. Beaumont, E., Roch, E., Chopin, L. & Roingeard, P. Hepatitis C Virus E1 and E2 Proteins Used as Separate Immunogens Induce Neutralizing Antibodies with Additive Properties. *PLoS One* **11**, e0151626 (2016).

3. Beaumont, E., Patient, R., Hourioux, C., Dimier-Poisson, I. & Roingeard, P. Chimeric hepatitis B virus/hepatitis C virus envelope proteins elicit broadly neutralizing antibodies and constitute a potential bivalent prophylactic vaccine. *Hepatology* **57**, 1303–1313 (2013).

SUPPLEMENTARY FIGURE LEGENDS

**Supplementary Figure S1.** Schematic representations of the chimeric HBV-HCV envelope proteins. The full-length genotype 1a HCV envelope proteins E1 and E2 are represented in green and red, respectively, while the HBV S protein (subtype *adw*), deleted from its first transmembrane domain (ΔTMD1), appears in cyan. TMDs are illustrated as boxes inside the proteins. In the E1 protein, the putative fusion peptide (FP) region is indicated. In the E2 protein, the main regions are as well represented: hypervariable regions (HVR1 and HVR2), the intergenotypic variable region (igVR), the front and back layers, the CD81 binding loop (CD81bl) and the stem region. N-glycosylation sites are represented in red above the proteins.

Supplementary Figure S2. Evaluation of the interactions between apoE and HBV S or HCV envelope proteins through co-immunoprecipitation assays. (a) Co-immunoprecipitation (co-IP) experiment between apoE and HBV S protein. Western-blot analysis of BHK-21 cell lysates expressing β-gal or HBV S protein, detected with goat anti-S polyclonal antibody (pAb) (70-HG15) (left panel). Western-blot analysis of immune complexes from co-IP captured with anti-S pAb (70-HG15) or goat isotype, and detected with anti-S (70-HG5) and goat anti-apoE (AB947) pAbs (right panel). (b) Co-IP experiment between apoE and HCV envelope proteins. Western-blot analysis of BHK-21 cell lysates expressing HCV E1 or E2 envelope proteins, detected with the mouse anti-E1/anti-E2 monoclonal antibodies (mAbs) (A4 and H52) (left panel). Western-blot analysis of immune complexes from co-IP with anti-apoE pAb (AB947) or goat isotype, and detected with anti-E1/anti-E2 mAbs (A4 and H52) and anti-apoE pAb (AB947) (right panel).

Supplementary Figure S3. Evaluation of the expression levels of proteins of interest of CHO cell clones. Lysates from clones stably expressing proteins of interest (S, E1-S, E2-S and apoE) were analyzed by western blotting. Proteins of interest were detected with mouse anti-E1 (A4), and anti-E2 (H52) mAbs, and goat anti-S (70-HG15) and anti-apoE (AB947) pAbs.

**Supplementary Figure S4.** Neutralization of HCV in cell culture with rabbit serum samples collected at day 42. Rabbit serum samples (collected on days 0 and 42), with a 1:5 dilution, were incubated with HCV in cell culture (HCVcc) harboring envelope proteins from genotype 1a (H77) or 2a (JFH1) isolates, which were then used to infect Huh7.5 cells. Infection levels were determined 48 hours post-infection, in a focus-forming unit staining assay. The % neutralization Δ(D42-D0) was determined by subtracting the % neutralization obtained with the pre-immune serum (day 0) from the one obtained with the post-immune serum (day 42) from the same rabbit. The assay was performed once in triplicate, and the results are expressed as mean values. Each colored point represents the % neutralization value calculated for a serum sample from an individual immunized rabbit, whereas the horizontal line represents the median value. The color code established for the antibody responses (Fig. 4), one color per rabbit, was conserved to simplify the correlation of results from both experiments.

Supplementary Figure S5. Neutralizing potential of antibodies induced by the immunization of rabbits with purified HBV-HCV subviral particles containing or not apoE, against genotype 2a HCVcc. (**a**) % neutralization of genotype 2a HCVcc. Rabbit serum samples (collected on days 0 and 56), with a 1:5 dilution, were incubated with HCVcc harboring envelope proteins from a genotype 2a isolate (JFH1), which were then used to infect Huh7.5 cells. Infection levels were determined 48 hours post-infection, in a focus-forming unit staining assay. The % neutralization Δ(D56-D0) was determined by subtracting the % neutralization obtained with the pre-immune serum (day 0) from the one obtained with the post-immune serum (day 56) from the same rabbit. The assay was performed once in triplicate, and the results are expressed as mean values. Each colored point represents the % neutralization value calculated for a serum sample from an individual immunized rabbit, whereas the horizontal line represents the median value. The color code established for the antibody responses (Fig. 4), one color per rabbit, was conserved to simplify the correlation of results from both experiments. **(b)** Analysis of the neutralizing capacity of anti-E2 antibodies for the best four rabbits (% neutralization > the median) of each group (S+E2-S versus S+E2-S+apoE), in terms of antibody level, as evaluated by ELISA, on day 56. Results are expressed here as the ratio of the change in % neutralization between days 0 and 56 (∆(D56-D0)) to the OD (490 nm) of anti-E2 antibodies obtained by ELISA on day 56 (Fig.4). The groups were compared in non-parametric Mann-Whitney *U* test. (*) *p*<0.05

**Supplementary Figure S6.** Evaluation of the neutralizing properties against HCVcc of serial serum dilutions of the two most reactive antisera of each group. Five-fold serial dilutions (1/5, 1/25, 1/125 and 1/625) of rabbit sera collected on days 0 and 56 were first incubated with HCVcc harboring envelope glycoproteins derived from genotype 1a (H77) and 2a (JFH1) isolates for 1 hour at 37°C, which were then used to infect Huh7.5 cells for 6 hours. Infection levels were determined after 48 hours of incubation at 37°C, in a focus-forming staining assay. The % neutralization Δ(D56-D0) was determined by subtracting the % neutralization obtained with the pre-immune serum (day 0) from the one obtained with the post-immune serum (day 56) from the same rabbit. The assay was performed once in triplicate, and the results are expressed as mean values. Each colored point represents the % neutralization value calculated for a serum sample from an individual immunized rabbit.

FULL GEL PICTURES

Fig. 1

Fig. 1a. BHK-21 cell lysates (co-expression)

**
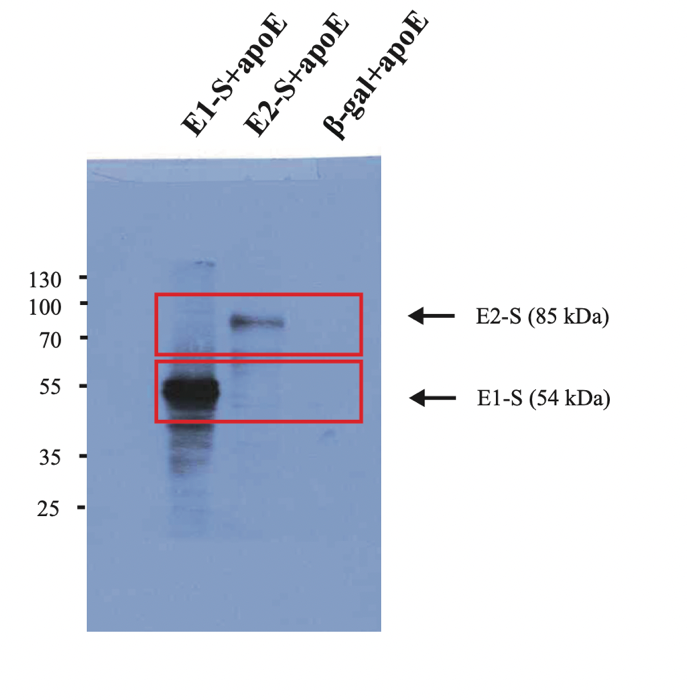
**Anti-E1 (A4) and anti-E2 (H52)

**
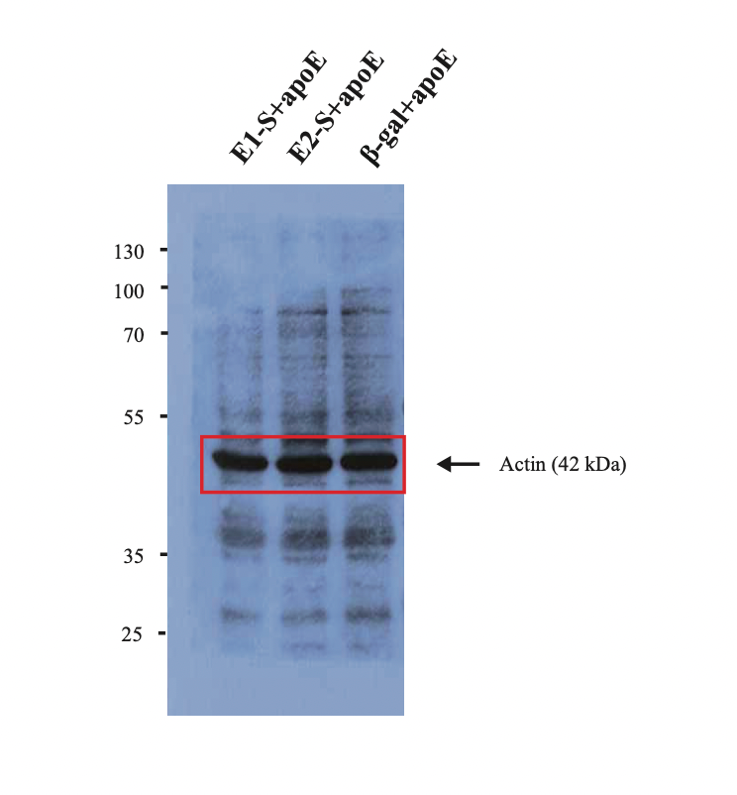
**Anti-actin (**A1978)**

**
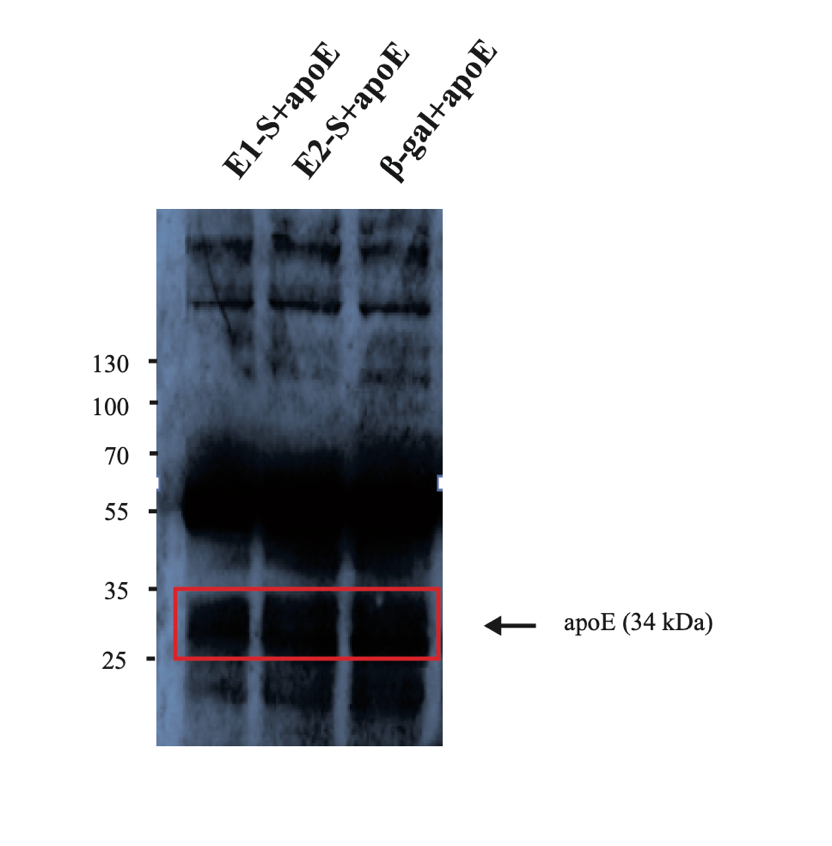
**Anti-apoE (AB947)

Fig. 1b. Co-immunoprecipitation

Anti-E1 (A4)

**
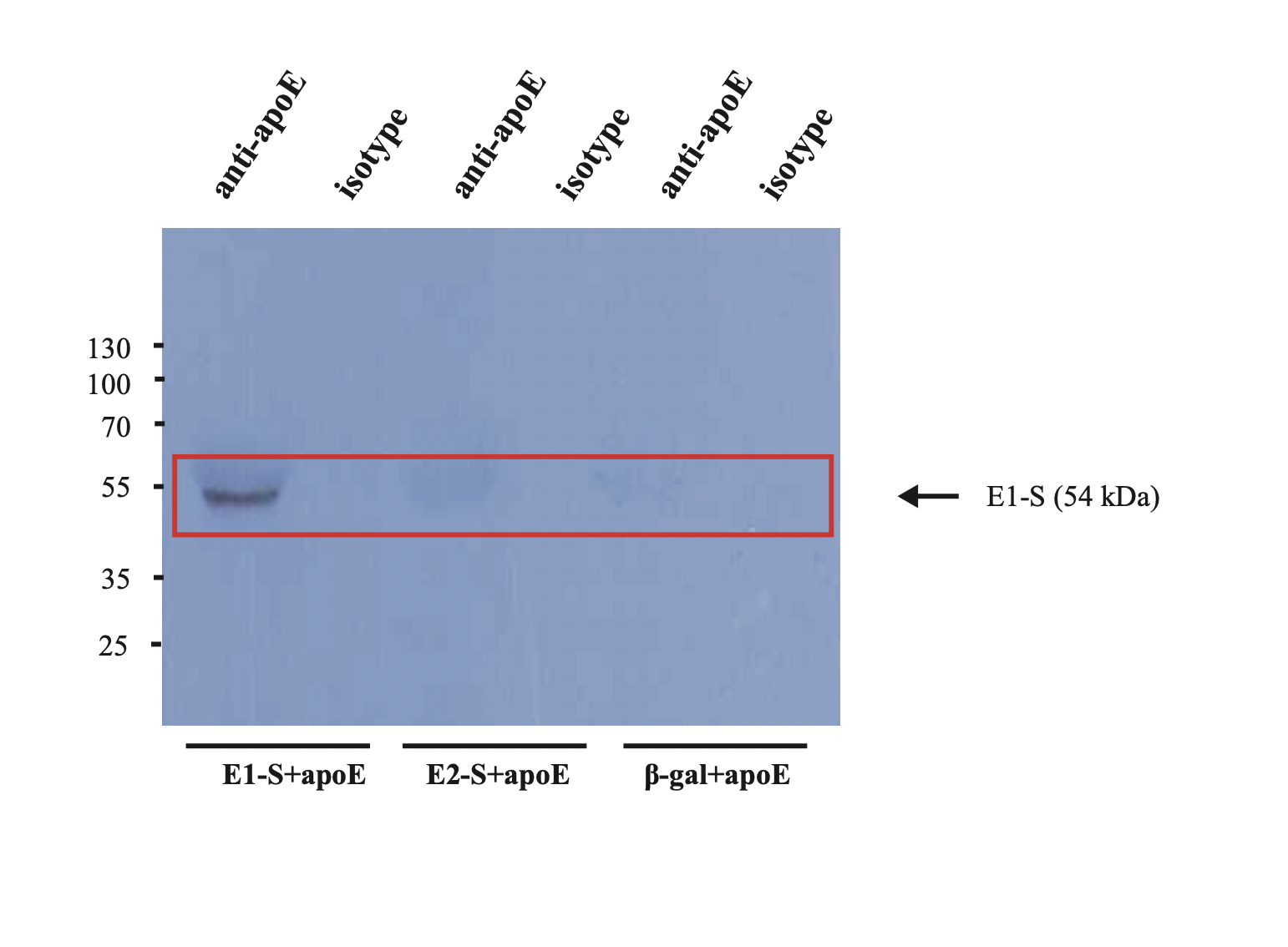
**

Anti-E2 (H52)

**
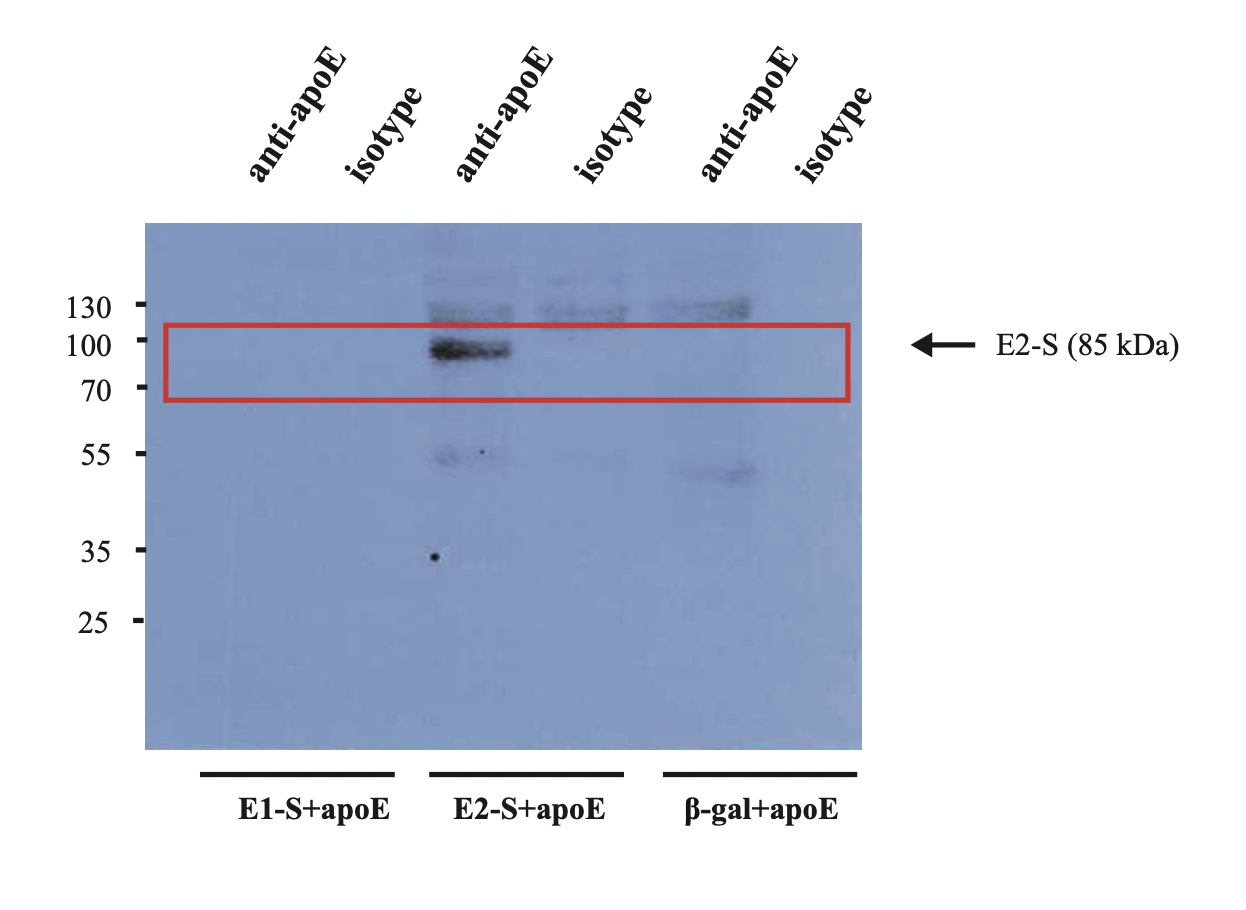
**

Anti-apoE (AB947)

**
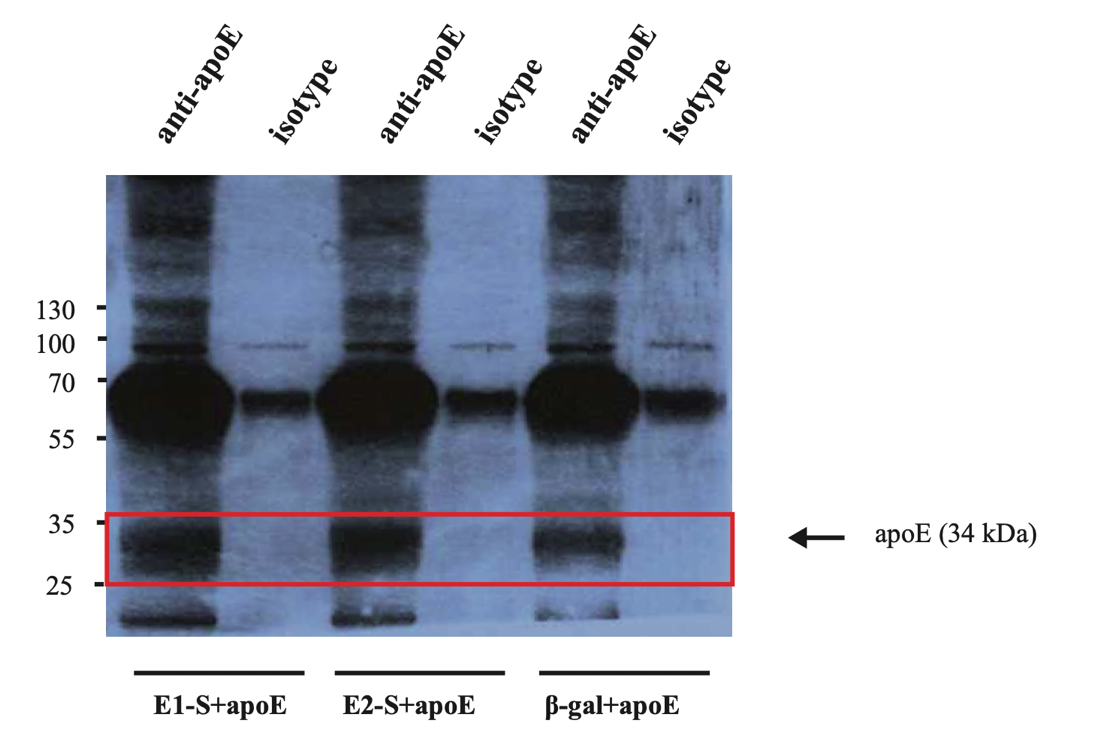
**

**
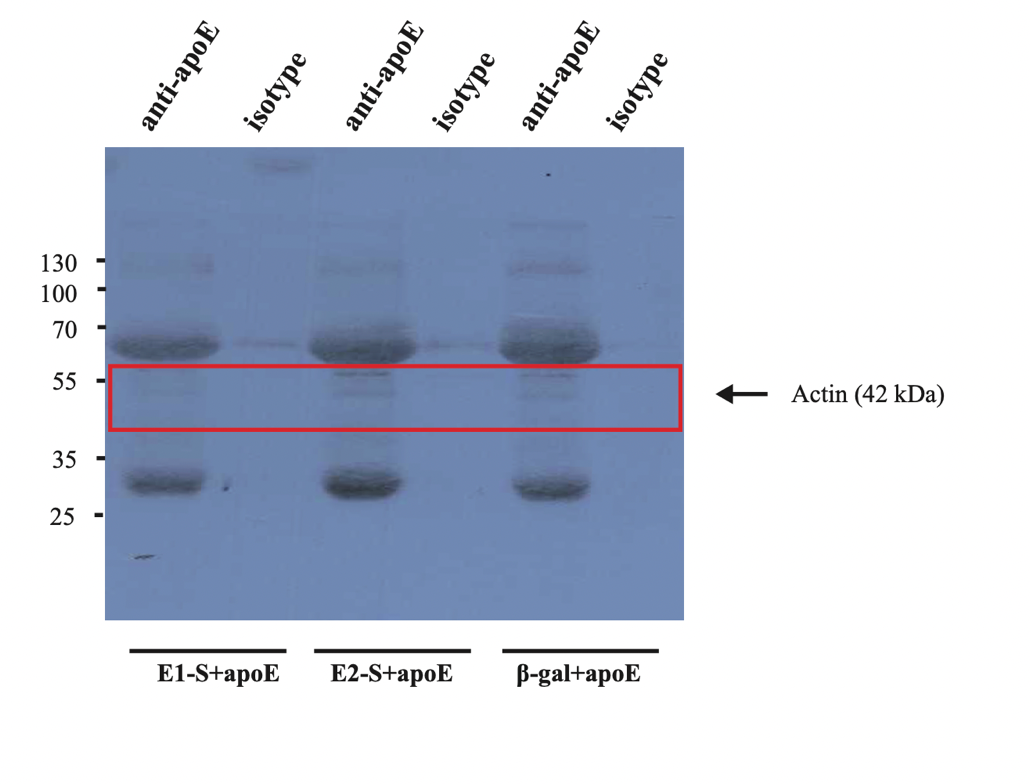
**Anti-actin (A1978) (after stripping of the membrane stained with anti-apoE)

Fig. 2a

**Anti-S (70-HG15)**

**
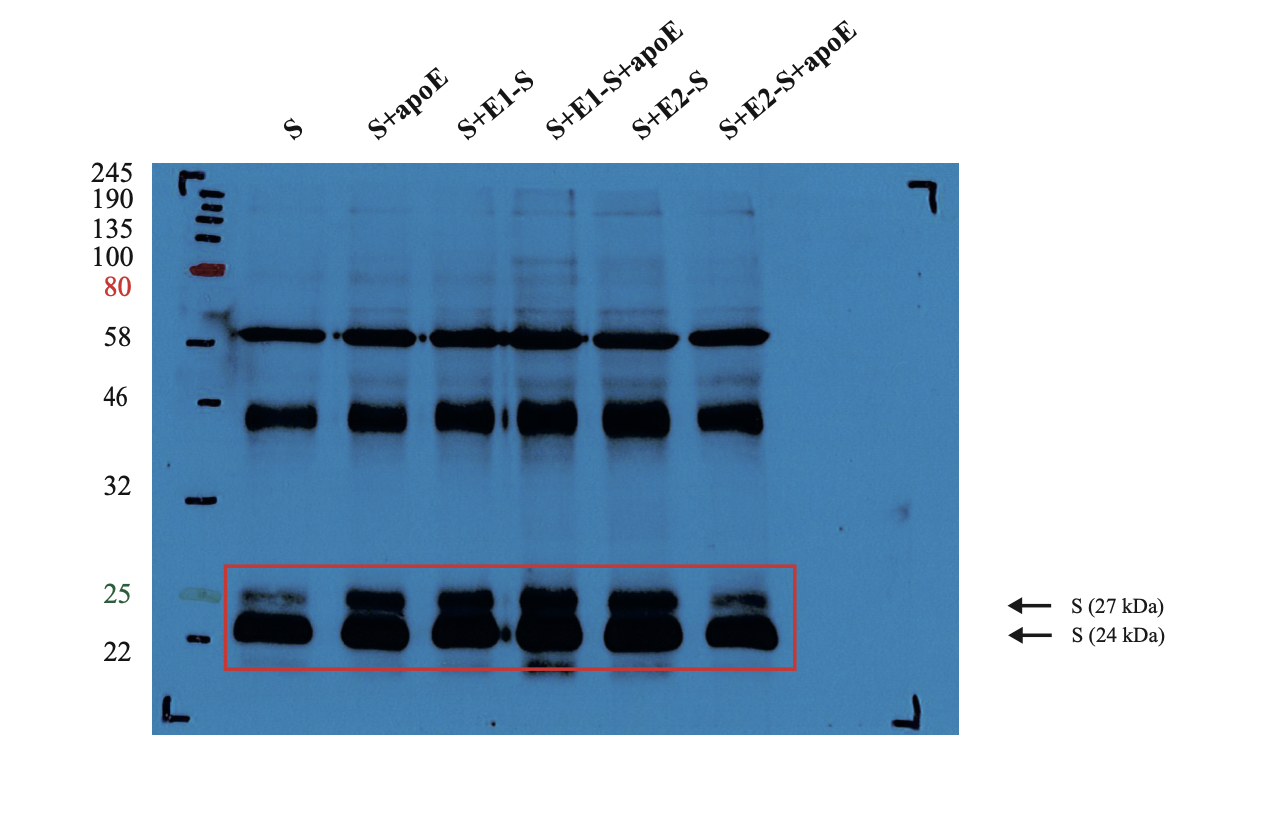
**

**Anti-E1 (A4)**

**
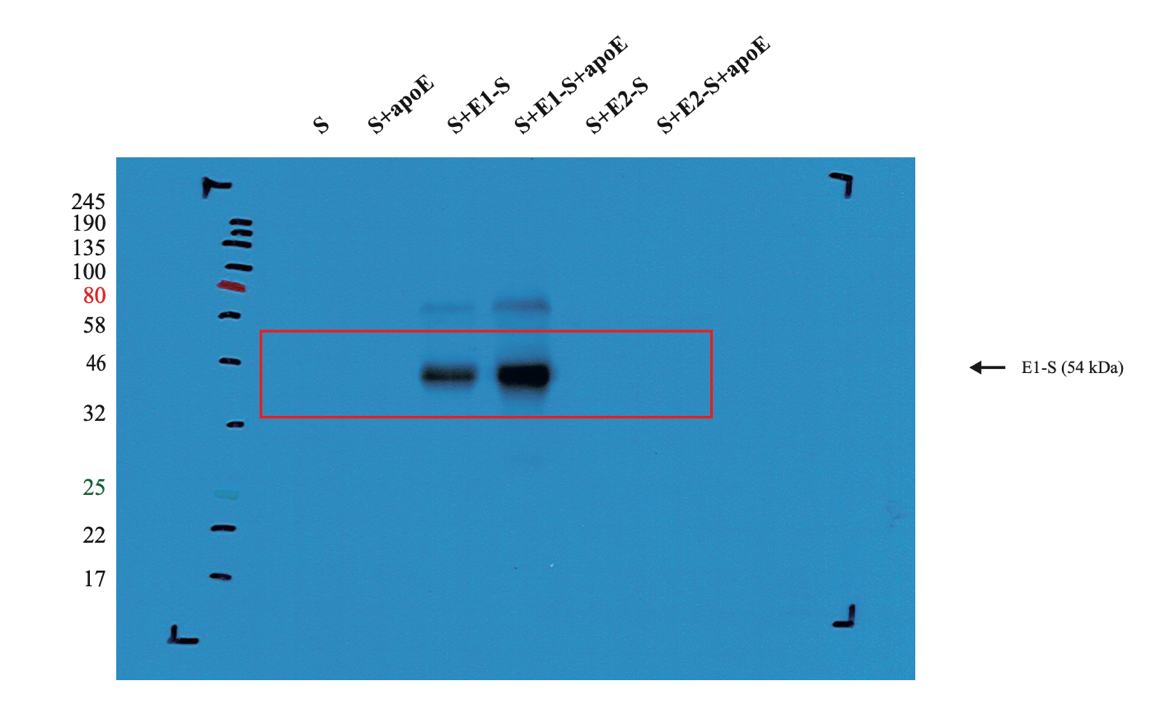
**

**
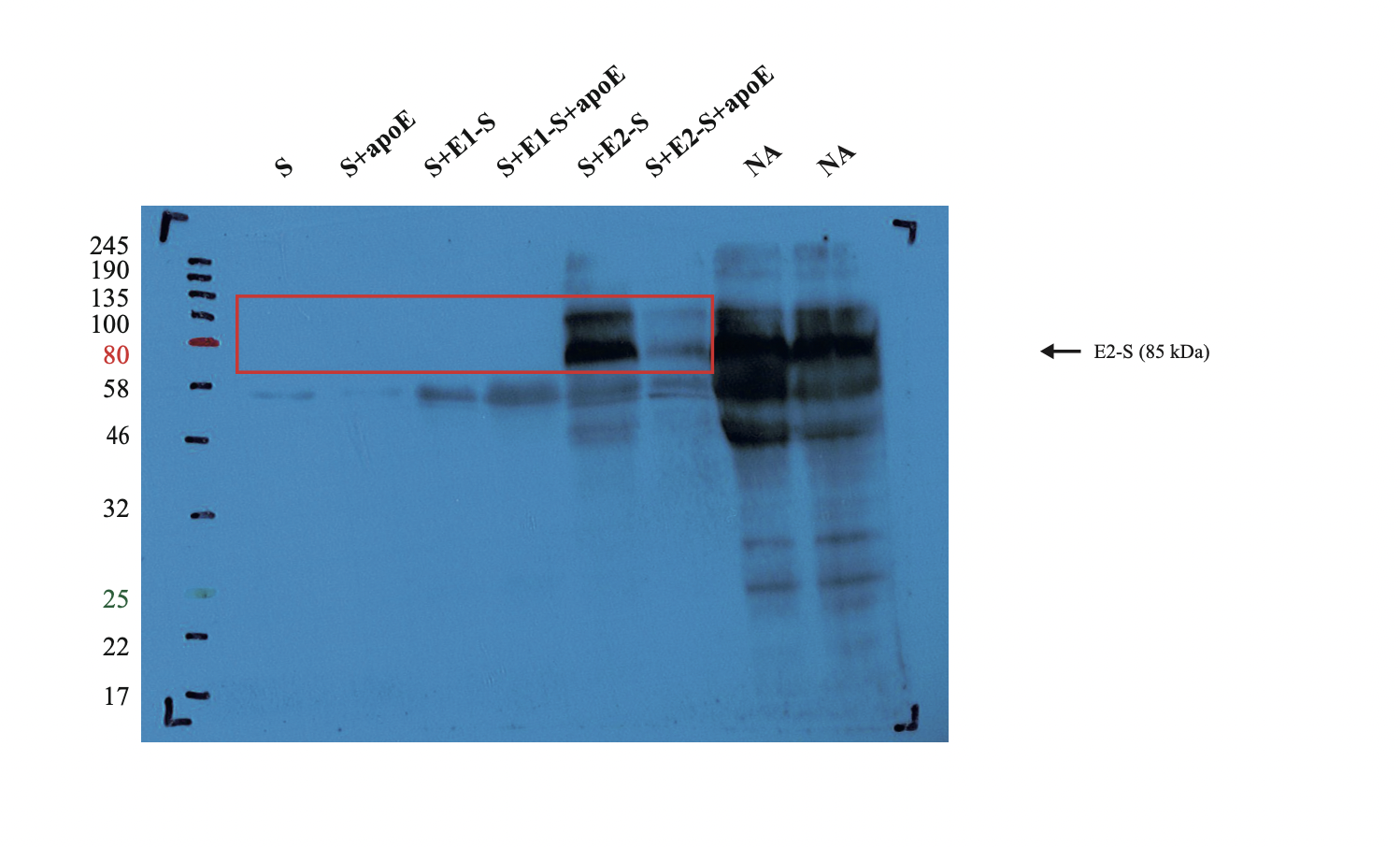
Anti-E2 (H52)**

**
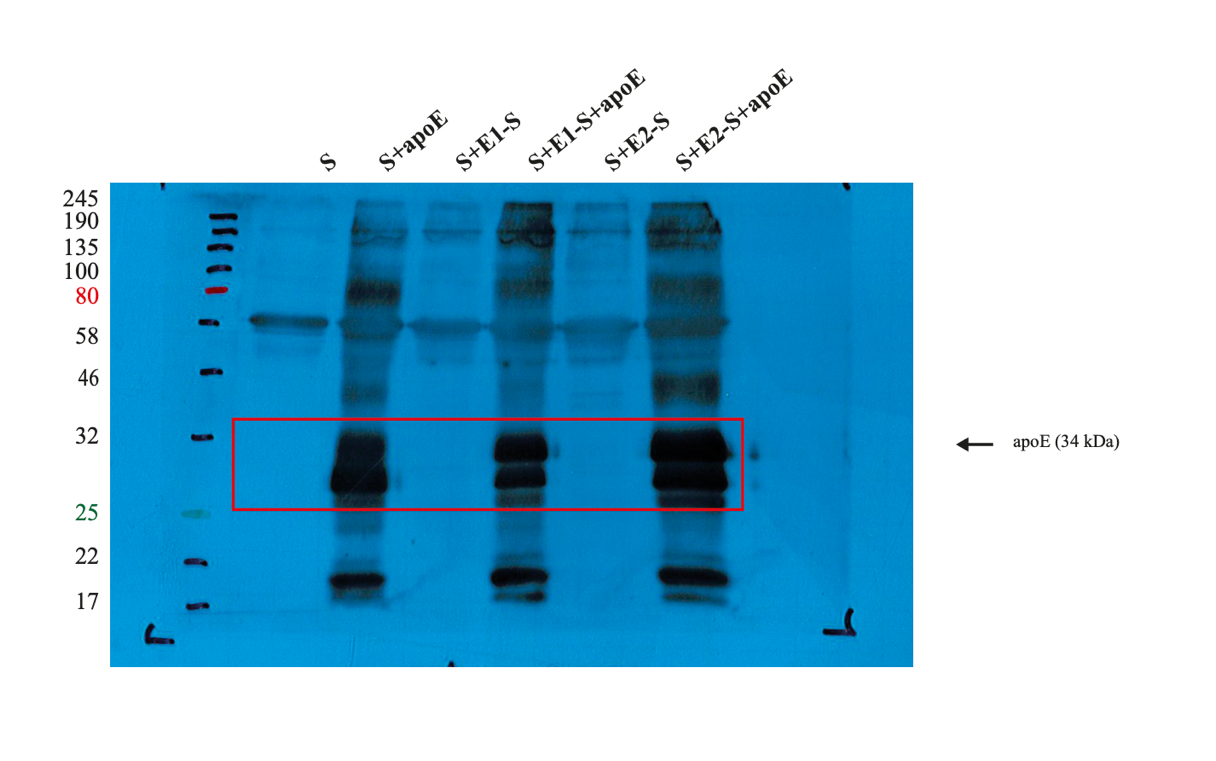
Anti-apoE (AB947)**

**Supplementary Fig. S2**

Supplementary Fig. S2a

**
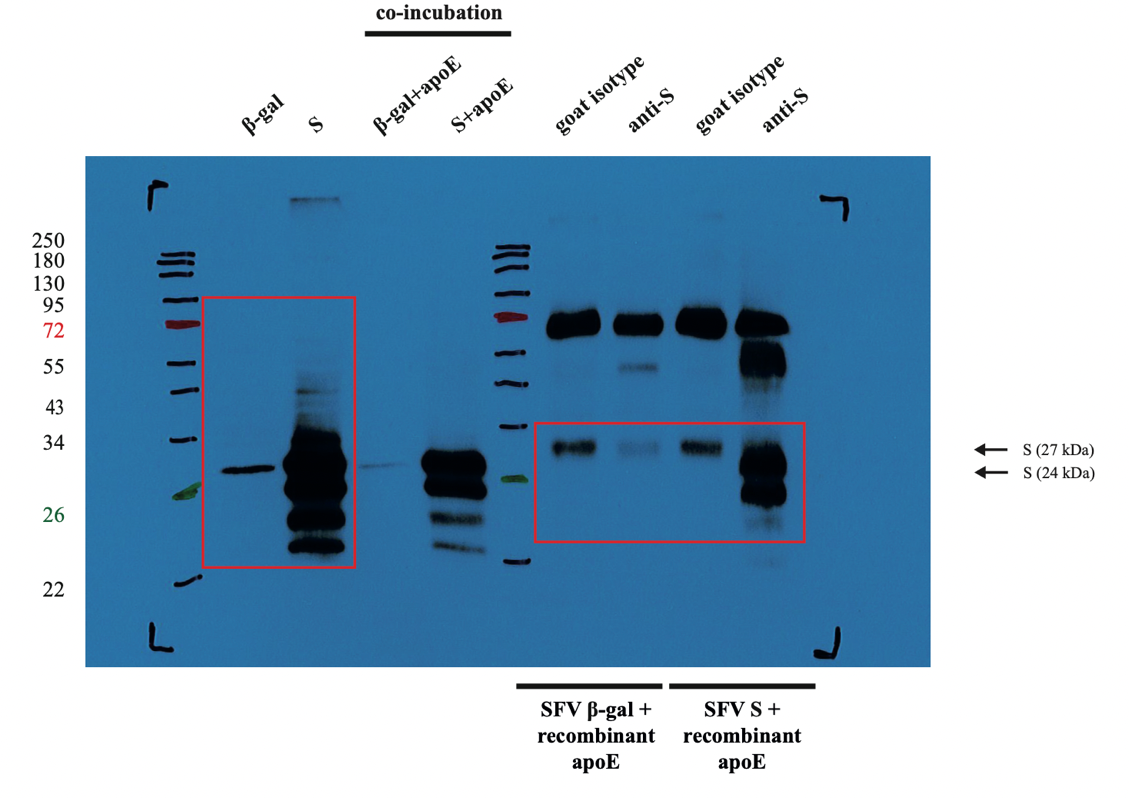
Anti-S (70-HG15)**

**
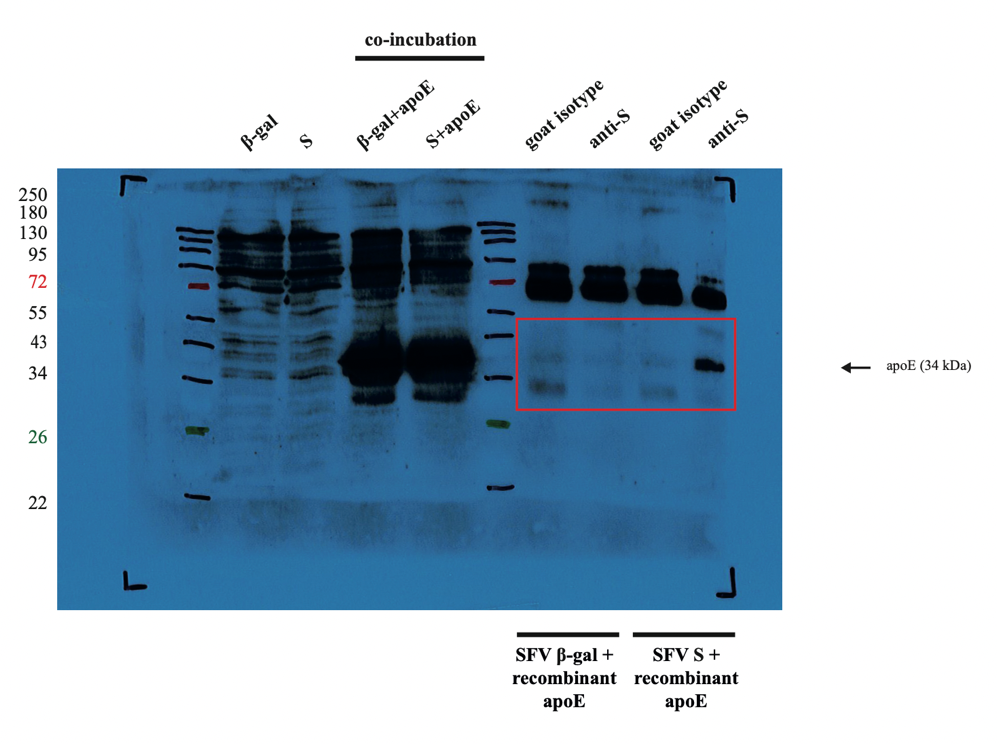
Anti-apoE (AB947)**

Supplementary Fig. S2b

BHK-21 cell lysates

**
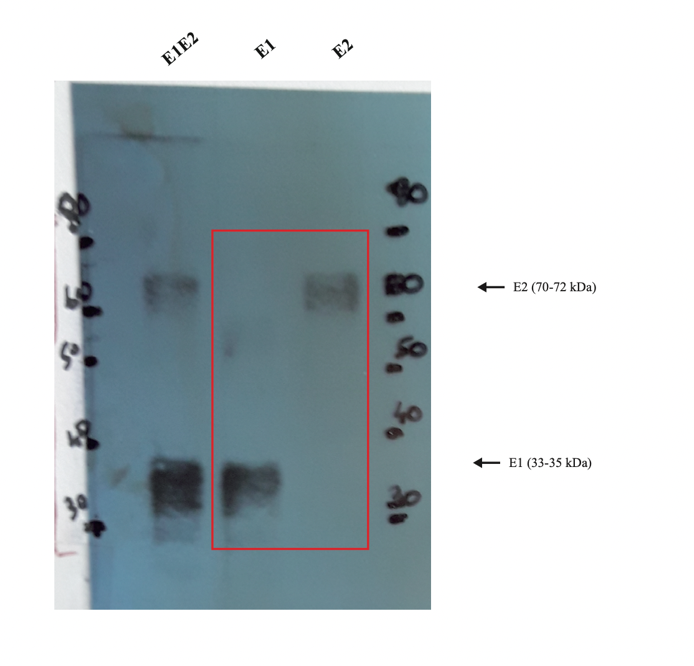
Anti-E1 (A4) and anti-E2 (H52)**

Co-immunoprecipitation

**Anti-apoE (AB947)**

**
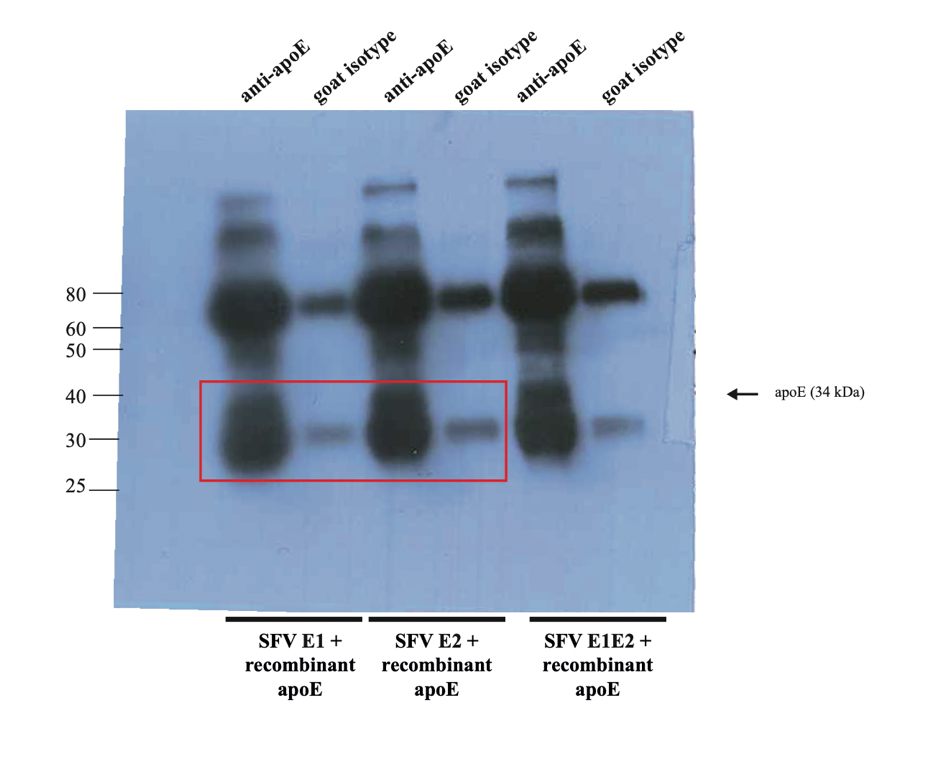
**

**
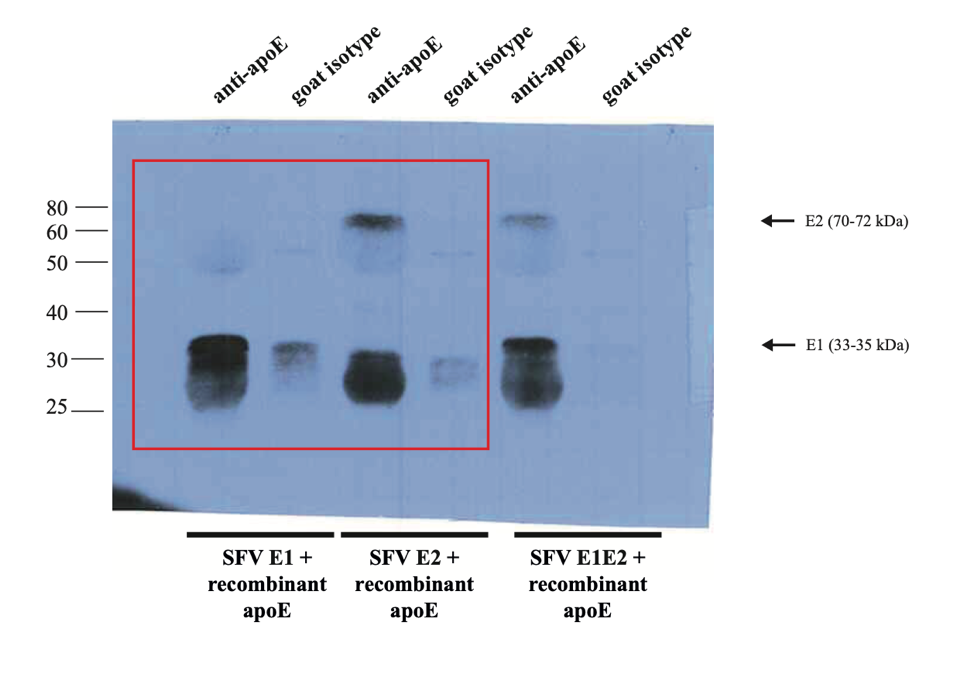
Anti-E1 (A4) and anti-E2 (H52)**

**Supplementary Fig. S3**

**
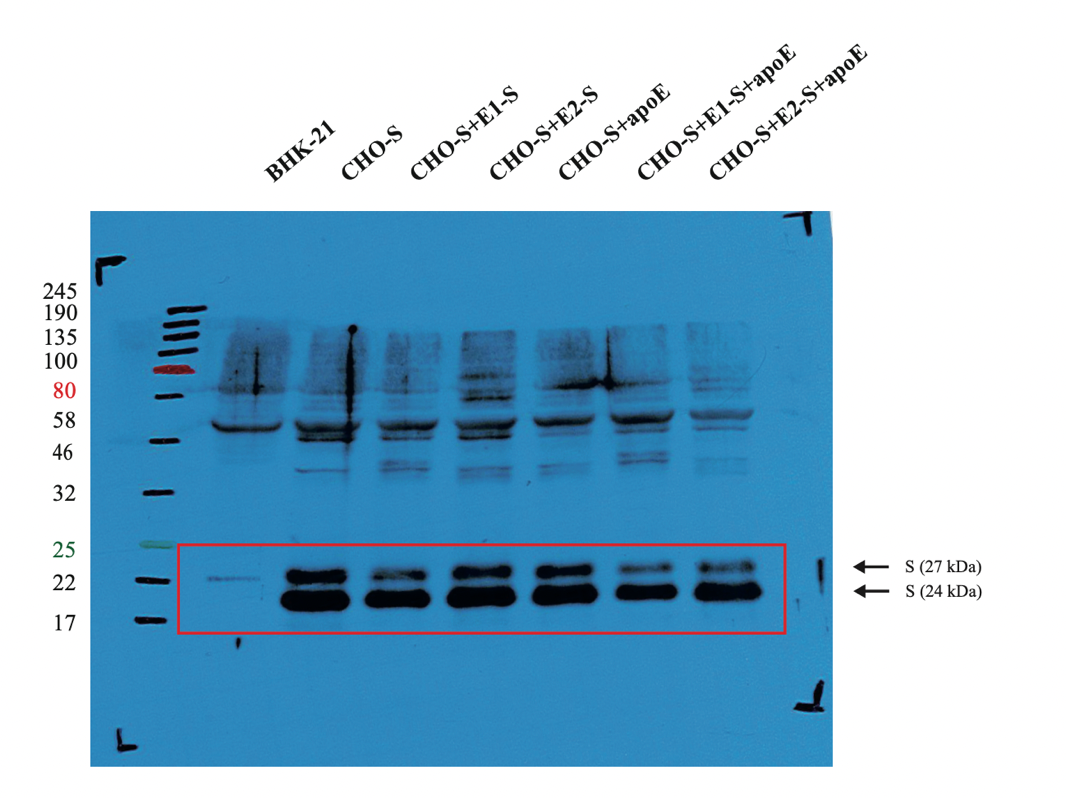
Anti-S (70-HG15)**

**
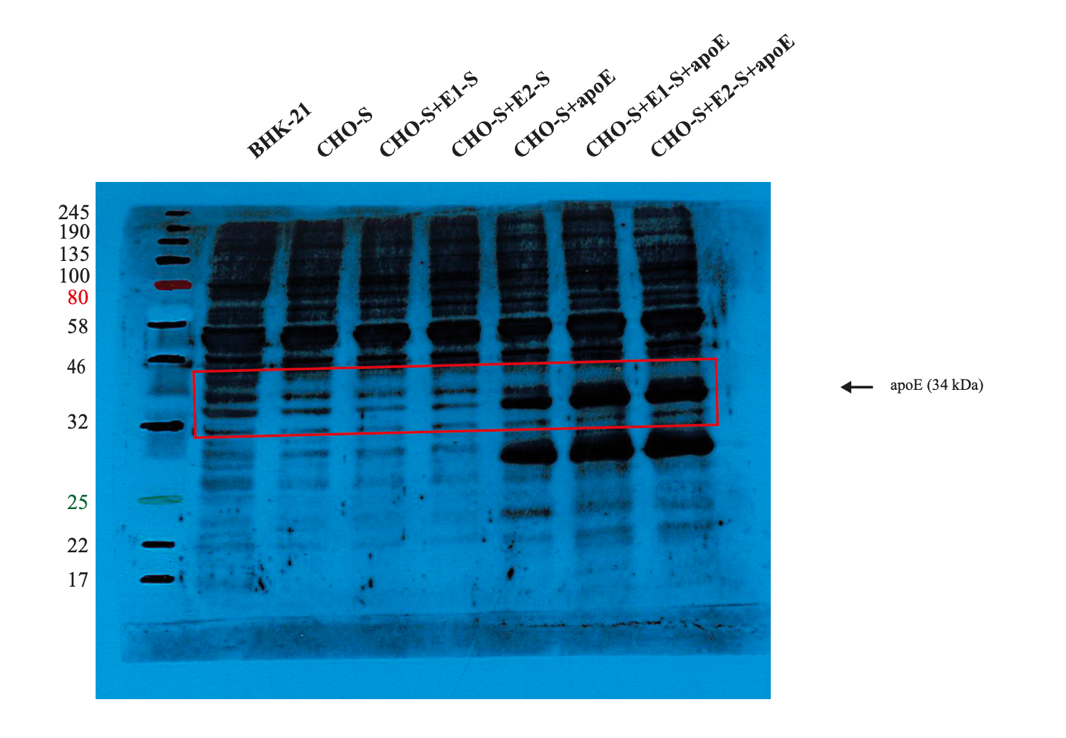
Anti-apoE (AB947)**

**Anti-E1 (A4) and anti-E2 (H52)**

**
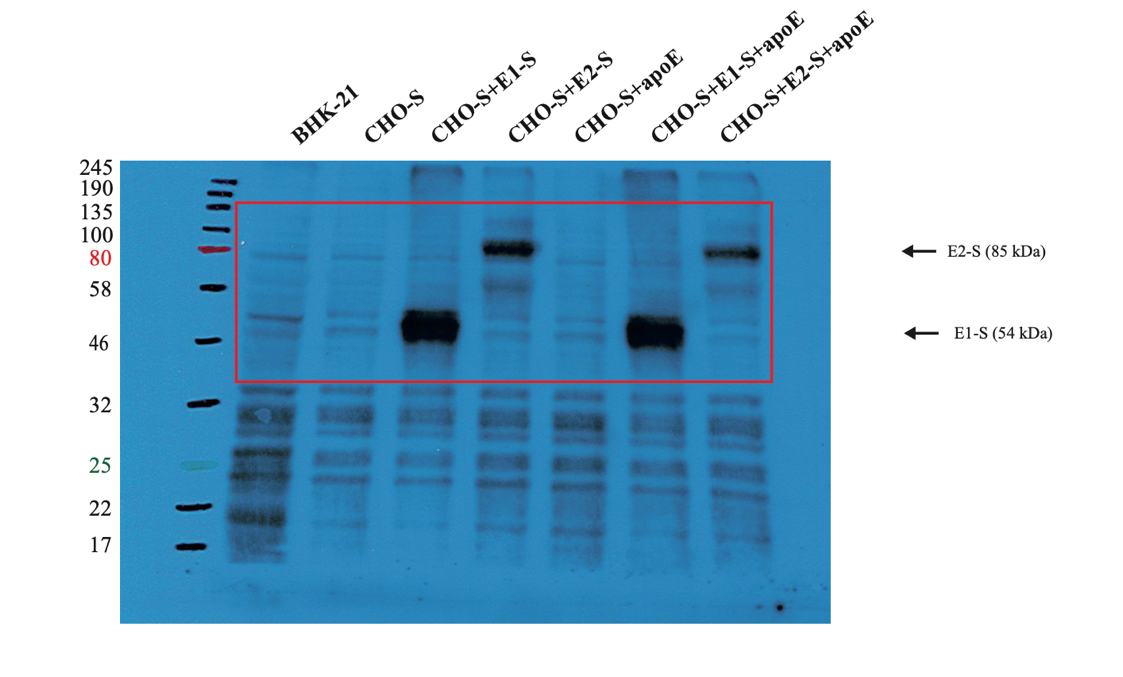
**
